# Author Correction: CelFiE-ISH: a probabilistic model for multi-cell type deconvolution from single-molecule DNA methylation haplotypes

**DOI:** 10.1186/s13059-024-03330-7

**Published:** 2024-07-08

**Authors:** Irene Unterman, Dana Avrahami, Efrat Katsman, Timothy J. Triche, Benjamin Glaser, Benjamin P. Berman

**Affiliations:** 1 Department of Developmental Biology and Cancer Research, Institute for Medical Research Israel-Canada, Faculty of Medicine, The Hebrew University of Jerusalem, Jerusalem, Israel; 2 Department of Endocrinology and Metabolism, Hadassah Medical Center and Faculty of Medicine, Hebrew University of Jerusalem, Jerusalem, Israel; 3 Center for Epigenetics, Van Andel Research Institute, Grand Rapids, MI USA

**Correction** **:** **Genome Biol 25, 151 (2024)**

**https://doi.org/10.1186/s13059-024-03275-x**

Following publication of the original article [1], the authors identified an error in the author name of Timothy J. Triche Jr. The given name and family name were erroneously transposed.

The incorrect author name is: Triche Tim Jr.

The correct author name is: Timothy J. Triche Jr.

The author group has been updated above and the original article [1] has been corrected.
