# Supplementary material for: CelFiE-ISH: a probabilistic model for multi-cell type deconvolution from single-molecule DNA methylation haplotypes
Source: Genome Biol. 2024 Jun 10;25:151. doi: 10.1186/s13059-024-03275-x (PMC11163775; doi:10.1186/s13059-024-03275-x)
Supplement: Supplementary file 1 — Additional file 1. Supplementary figures S1-S13. [file 13059_2024_3275_MOESM1_ESM.docx]

# Additional file 1: supplementary figures


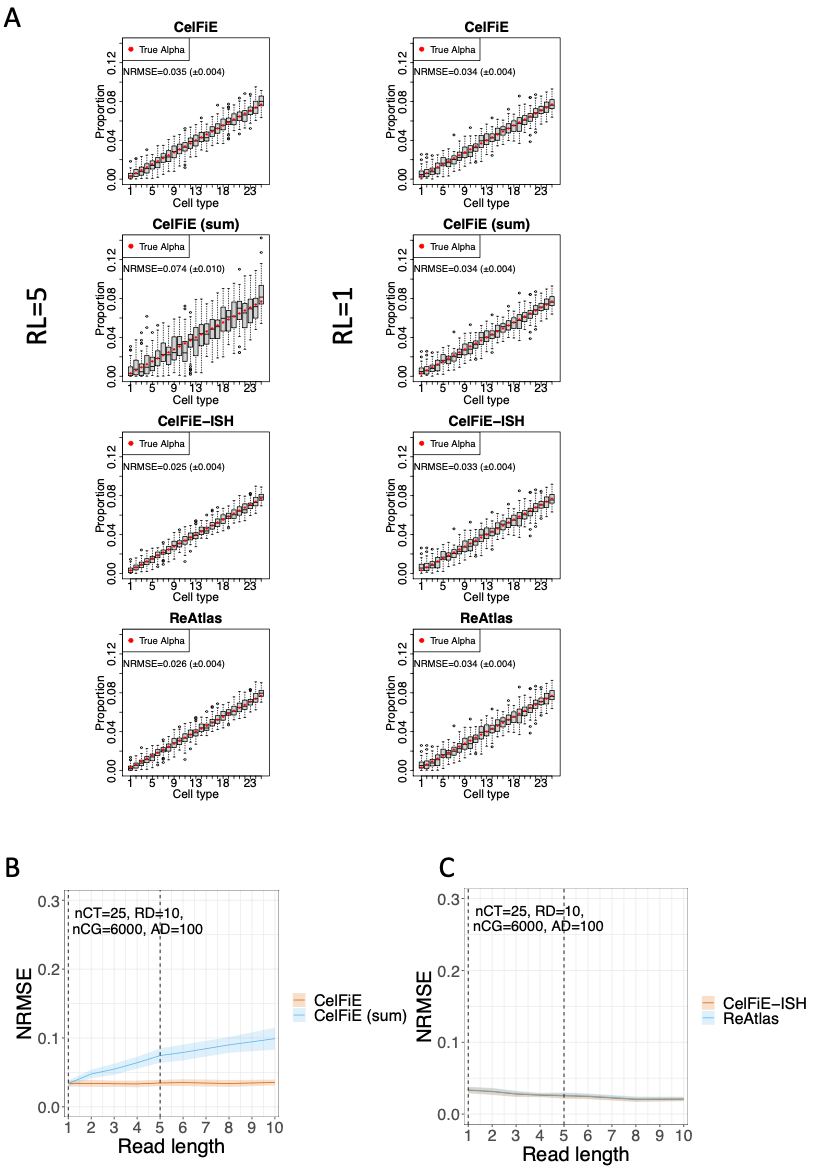


### **Figure S1: n-state simulations.** A) Estimated proportion of each cell type (gray box) vs. true proportion (red circle) for each model, with read length of 5 (left column) and 1 (right column). Each plot is based on 50 replicate simulations, and shows the Normalized Error Normalized error (Normalized Root Mean Square Error or NMRSE) and NRMSE IQR across replicates.

B, C) NRMSE of each model is shown across a range of read lengths. Shaded areas show standard deviation across 50 replicates. Vertical dotted lines show the condition from panel (A). CeFiE-Sum compared to CelFiE (B) and ReAtlas is compared to CelFiE-ISH (C). Since we kept the region size the same as the read length, with increasing region size the summed version of CelFiE loses information and performs poorly.

**
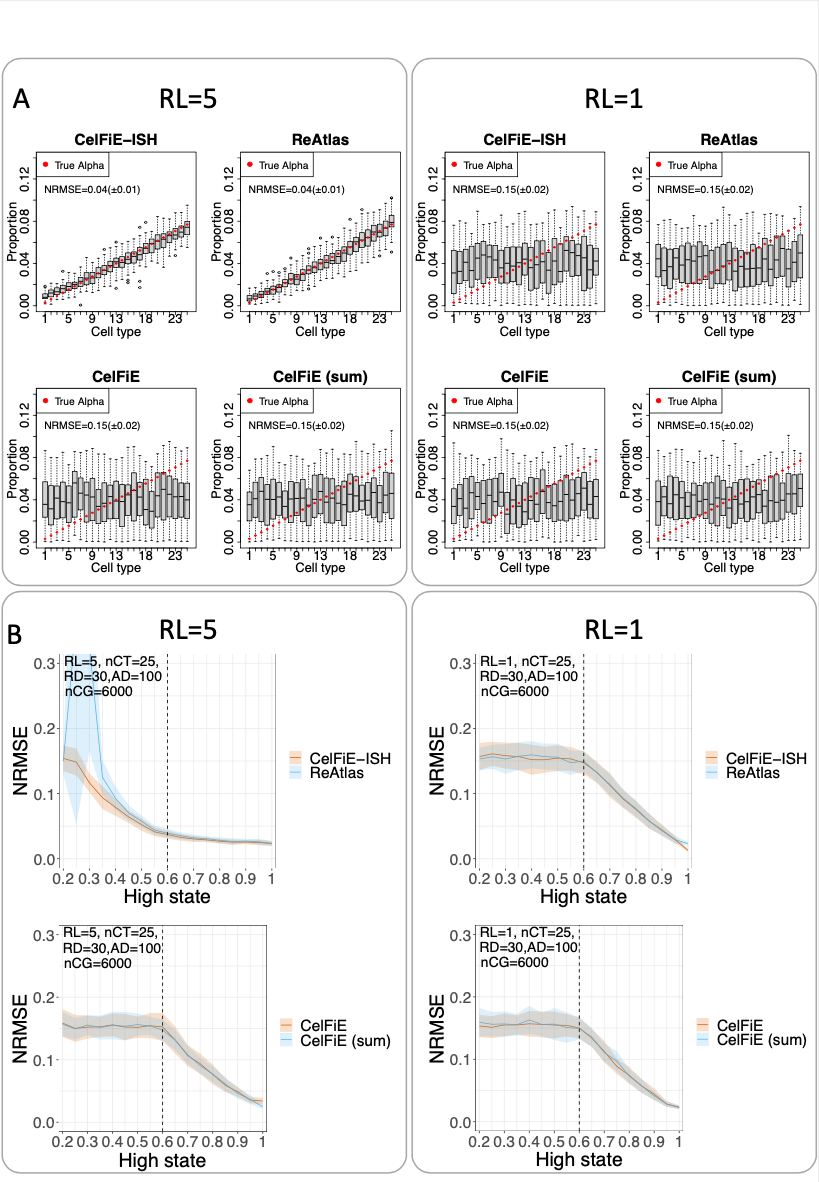
**

### **Figure S2: two-state simulations.** Simulations using [
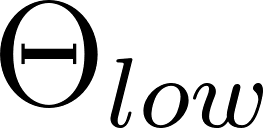
](https://www.codecogs.com/eqnedit.php?latex=%5CTheta_%7Blow%7D#0)=0.1 and [
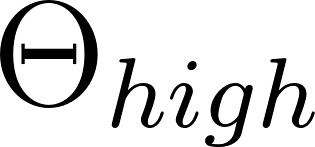
](https://www.codecogs.com/eqnedit.php?latex=%5CTheta_%7Bhigh%7D#0)=0.6, showing the estimated proportion of each cell type (gray box) vs. true proportion (red circle) for each model, using a read length of either RL=5 (left) or RL=1 (right). Each plot is based on 50 replicates simulations, and shows the Normalized Error Normalized error (Normalized Root Mean Square Error or NMRSE) and NRMSE standard deviation across replicates. (B) NRMSE as a result of varying the [
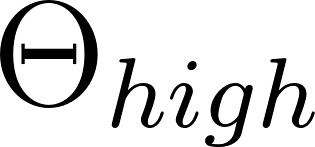
](https://www.codecogs.com/eqnedit.php?latex=%5CTheta_%7Bhigh%7D#0) state from 0.2 to 1.0 (with [
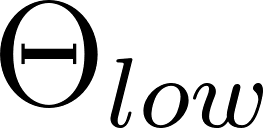
](https://www.codecogs.com/eqnedit.php?latex=%5CTheta_%7Blow%7D#0) held constant at0.1. Left using read length RL=5, and right using read length RL=1. Shaded area shows standard deviation across 50 replicates.A dotted vertical line shows the condition from panel A above. ReAtlas compared to CelFiE-ISH, CeFiE-Sum compared to CelFiE.


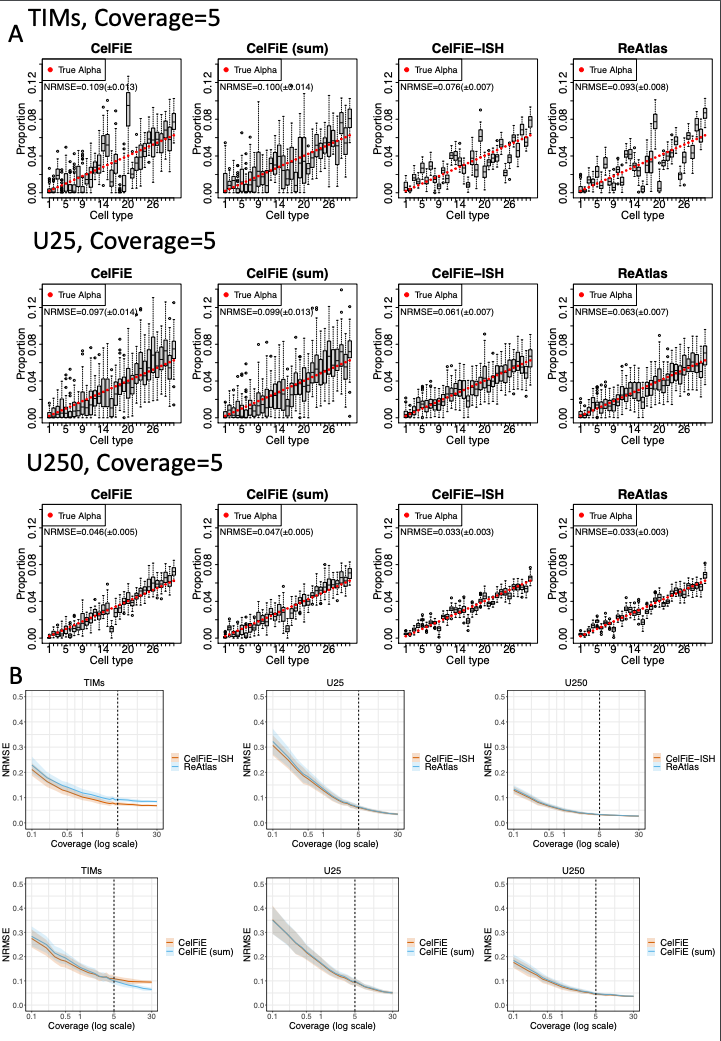


### **Figure S3: Performance of “Sum” and “Reatlas” model variants in high complexity in silico mixtures.** 31 cell types from the Loyfer et al. WGBS methylation atlas were mixed at set proportions to yield a total average read depth (coverage) of 5. (A) Deconvolution results using three different sets of input markers, Caggiano et al. method TIMs (top row), Loyfer et al. U25 (middle row), and Loyfer et al. U250 (bottom row). In each case, estimated proportions are shown as a boxplot of 50 replicates, and true mixture proportions are shown as a red circle. (B) Mixtures were performed for read depths (coverages) from 0.1x to 30x, showing NRMSE for each model and each marker set. Dotted vertical lines indicate the condition from panel A (coverage=5). Shaded areas show standard deviation across 50 replicates.

**
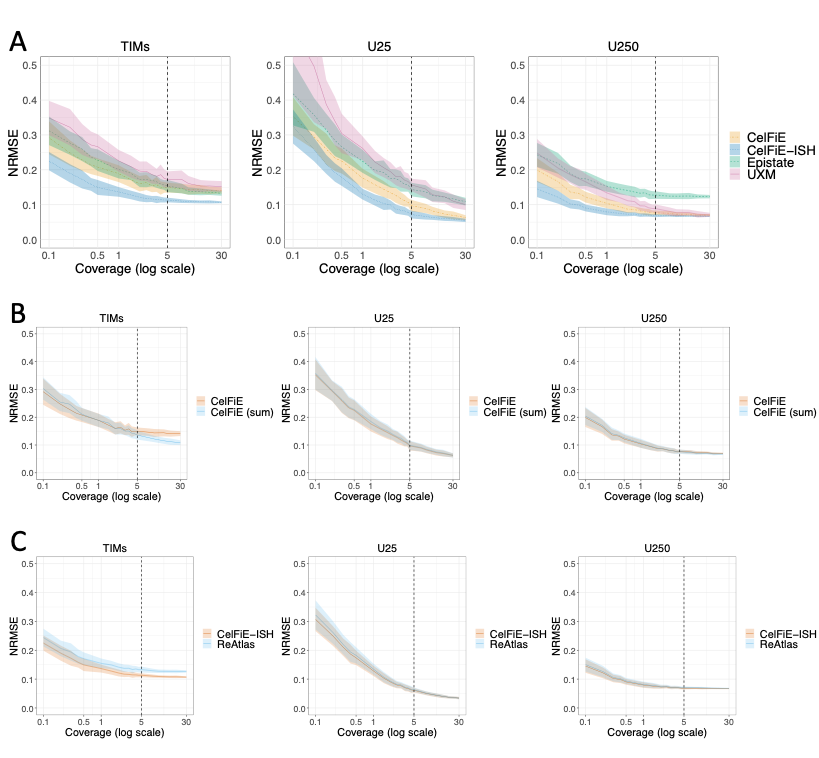
**

### **Figure S4: High complexity in silico mixtures with 2nd random cell type shuffling**, cell type randomly shuffled. A) NRMSE for each model and each marker set. Dotted vertical lines indicate the condition from figure 3 (coverage=5). B) effect of summing (sum-CelFiE) compared to CelFiE without summing. C) ReAtlas compared to CelFIE-ISH. The cell type order used is: Prostate-Ep, Pancreas-Beta, Lung-Ep-Alveo, Small-Int-Ep, Fallopian-Ep, Eryth-prog, Oligodend, Gastric-Ep, Breast-Basal-Ep, Breast-Luminal-Ep, Liver-Hep, Pancreas-Delta, Bladder-Ep, Blood-NK, Lung-Ep-Bron, Kidney-Ep, Thyroid-Ep, Blood-B, Heart-Cardio, Colon-Ep, Adipocytes, Pancreas-Alpha, Endothelium, Head-Neck-Ep, Blood-Granul, Blood-Mono+Macro, Neuron, Pancreas-Duct, Blood-T, Pancreas-Acinar, Ovary+Endom-Ep.

**
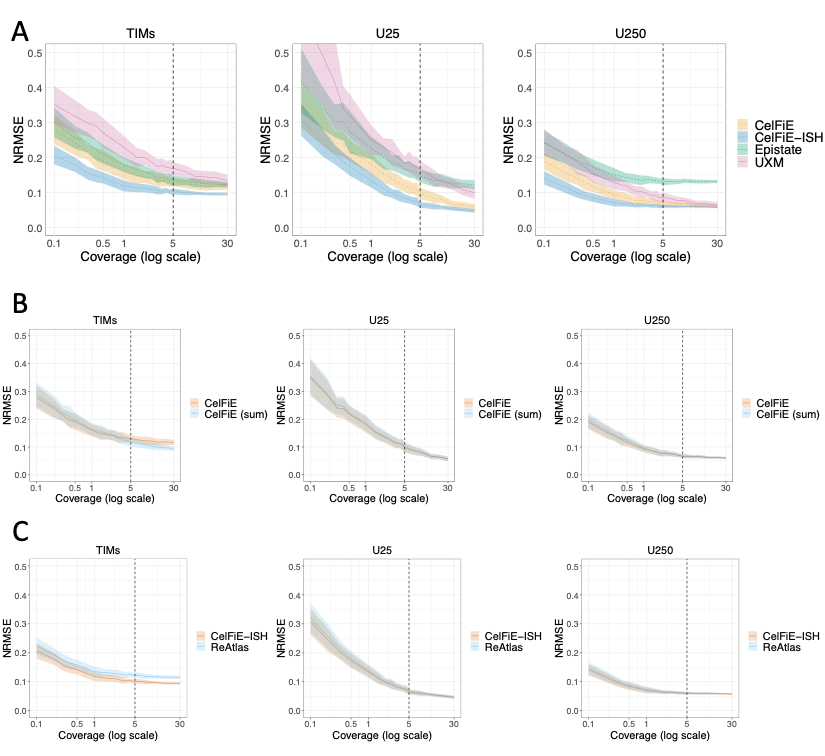
**

### **Figure S5: High complexity in silico mixtures with 3rd random cell type shuffling**, cell type randomly shuffled. A) NRMSE for each model and each marker set. Dotted vertical lines indicate the condition from figure 3 (coverage=5). B) effect of summing (sum-CelFiE) compared to CelFiE without summing. C) ReAtlas compared to CelFIE-ISH. The cell type order used is: Blood-NK, Head-Neck-Ep, Kidney-Ep, Blood-B, Small-Int-Ep, Breast-Luminal-Ep, Pancreas-Beta, Colon-Ep, Pancreas-Acinar, Gastric-Ep, Prostate-Ep, Breast-Basal-Ep, Eryth-prog, Liver-Hep, Oligodend, Blood-T, Neuron, Fallopian-Ep, Bladder-Ep, Pancreas-Delta, Blood-Granul, Thyroid-Ep, Endothelium, Lung-Ep-Bron, Lung-Ep-Alveo, Ovary+Endom-Ep, Heart-Cardio, Blood-Mono+Macro, Pancreas-Duct, Adipocytes, Pancreas-Alpha.


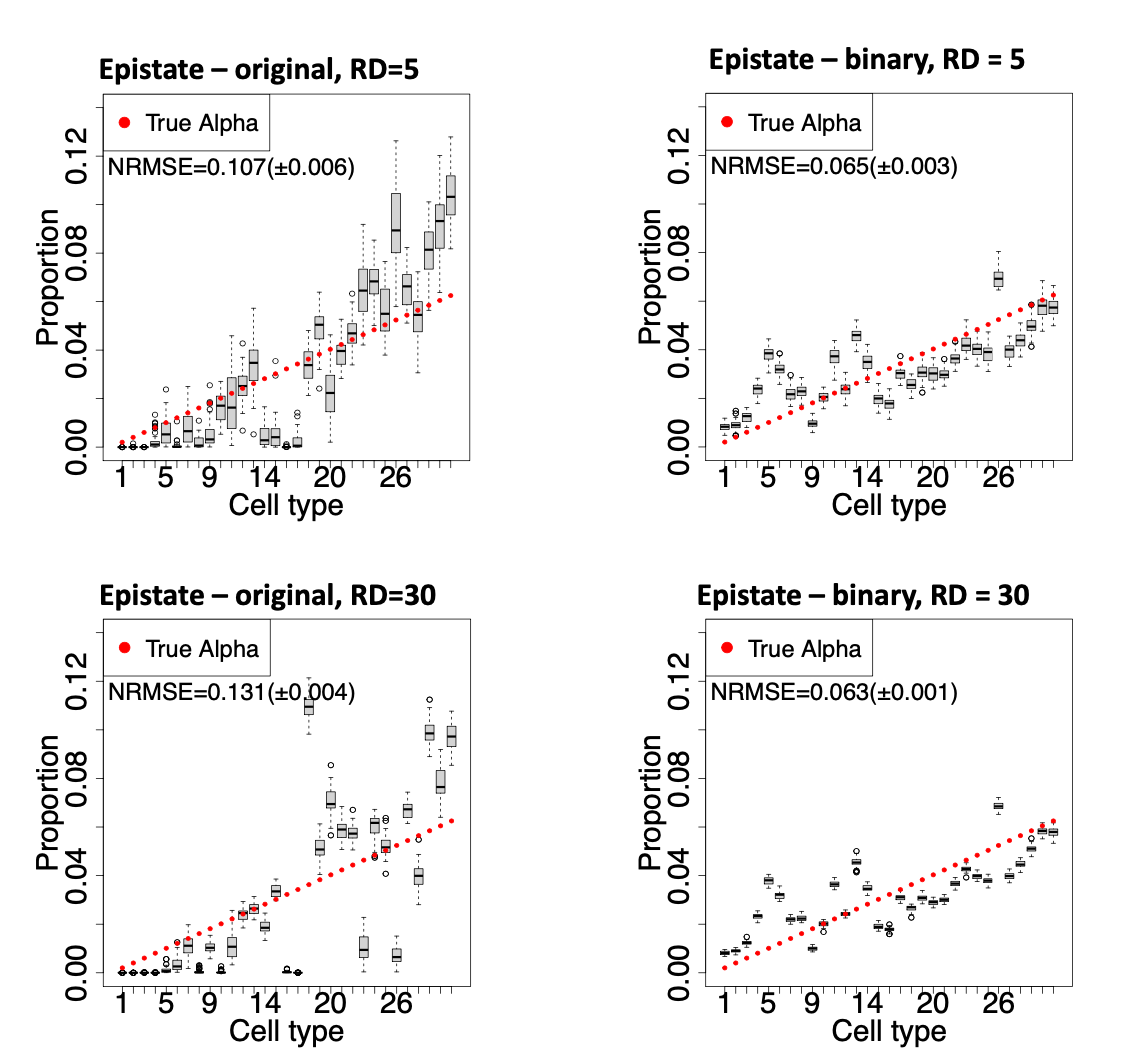


### **Figure S6: hard assignment of methylation states to cell types in Epistate.** Estimated proportions are shown for each of the 31 cell types as a boxplot of 50 replicates, and true mixture proportions are shown as a red circle. The cell type ordering is listed in the Methods. The default setting for Epistate is shown on the left, labeled “Epistate - original”. On the right is Epistate with hard assignment of each methylation state to a target cell type, on the same mixtures.

**
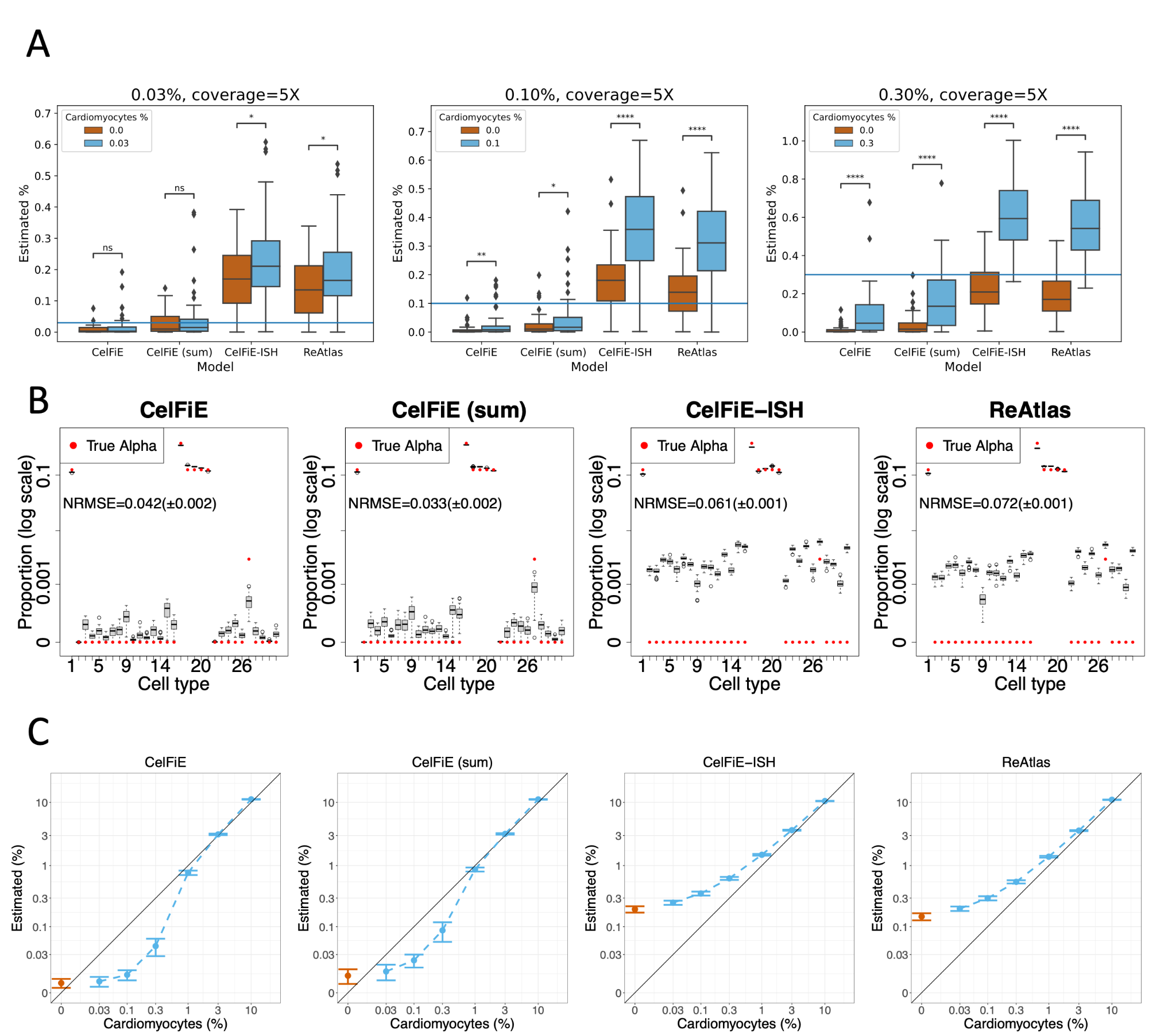
**

### **Figure S7: Performance of “Sum” and “Reatlas” model variants in Circulating DNA inspired in silico mixtures.** A) Cardiomyocyte samples were added to a background mixture of leukocytes and hepatocytes at varying proportions: 0.03% (left), 0.1% (middle) and 0.3% (right), at a depth of 5X. A one-tailed t-test was performed on the cardiomyocyte estimations of each model against a null mixture with no added cardiomyocytes. *<0.05, **<0.01, ***<0.001, ****<0.00001. B) Full 31-cell type deconvolution of 50 0.3% cardiomyocyte mixtures. In each case, estimated proportions are shown as a boxplot of 50 replicates, and true mixture proportions are shown as a red circle. To display the wide range of true proportions, the Y axis is in log scale.The cell type ordering is listed in the Methods. C) Mixtures of the leukocyte-hepatocyte background were performed for read depth 100,with 0-10% cardiomyocytes. Cardiomyocyte estimates across 50 replicates are shown, with standard deviation. Both axes are in log-scale.

**
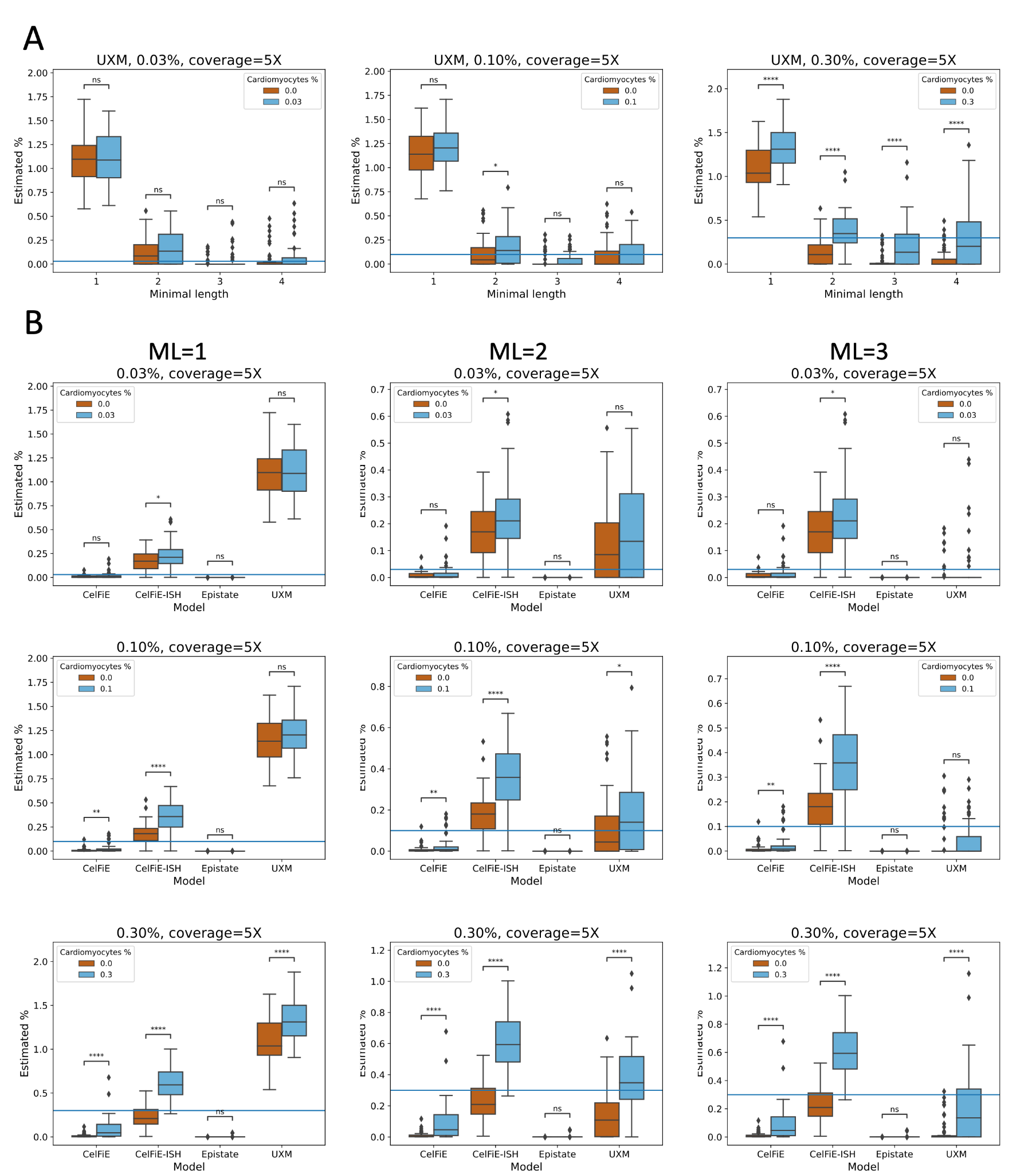
**

### **Figure S8: Performance of UXM minimal length adjustment in Circulating DNA inspired in silico mixtures**. The settings used for UXM in the Loyfer analyses was a minimum of 4 CpGs per fragment, and a threshold of 25% methylation for U fragments. However, these can be adjusted, and this could have implications on performance, especially in low-coverage settings, where the exclusion of a large proportion of reads could be detrimental to deconvolution. Here, we explore the effect of changing the minimal accepted number of CpGs per fragment (minimal length). The reference atlas remained constant at minimal 4 CpGs. A) UXM with varying thresholds was applied to the same spike-in mixtures shown in Figure 4A. B) UXM minimal length 1 (left), 2 (middle) and 3 (right) along with the other models from Figure 4A, at a target proportion of 0.03% (top), 0.1% (middle) and 0.3% (bottom).

###
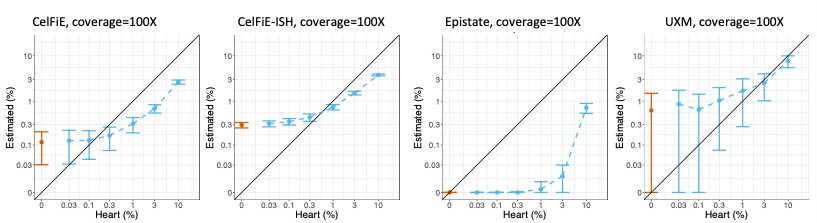
**Figure S9: Spike-In mixtures of RRBS tissue samples.** Heart RRBS samples were mixed at varying proportions into a background of WBC RRBS samples, and deconvoluted with the WGBS cell-type atlas, on U250 regions. Heart spike-in mixtures are shown in blue, compared to null mixtures with no heart reads (red points). Heart estimates across 50 replicates are shown as standard deviation bars. Both axes are in log-scale.


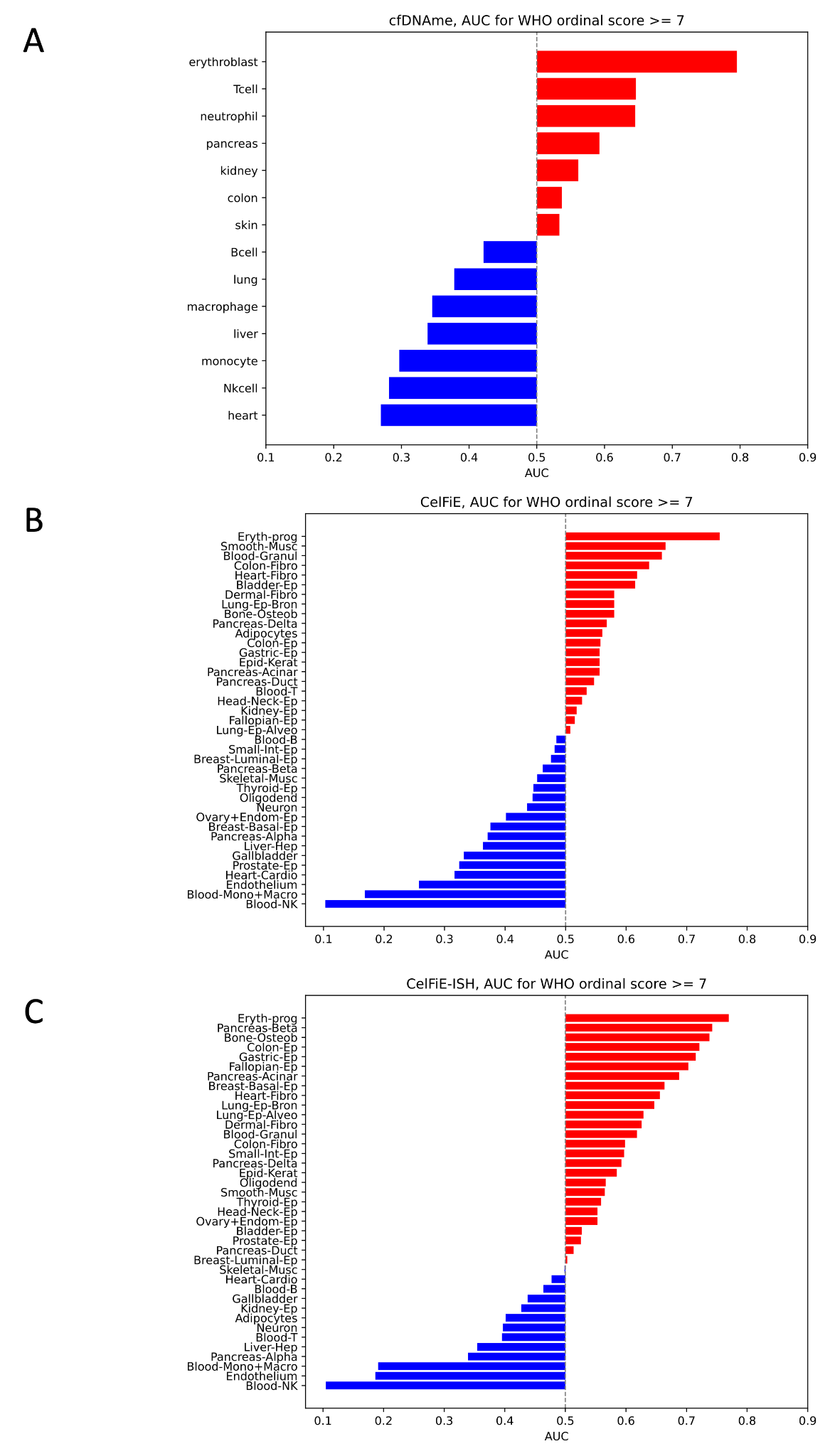


### **Figure S10: AUC scores for COVID-19 severity.** ROC analysis was performed using the proportions for each cell type separately to predict WHO ordinal code >= 7 (patients requiring mechanical ventilation in the ICU). Cell types are ranked by the AUC score. A) cfDNAme, fractions as reported in [[29]](https://paperpile.com/c/xgyw2e/PyoX). AUC of CelFiE (B) and CelFiE-ISH (C) fractions, based on the U250 regions and the 39-cell type Loyfer atlas [[1]](https://paperpile.com/c/xgyw2e/FaCh).

**
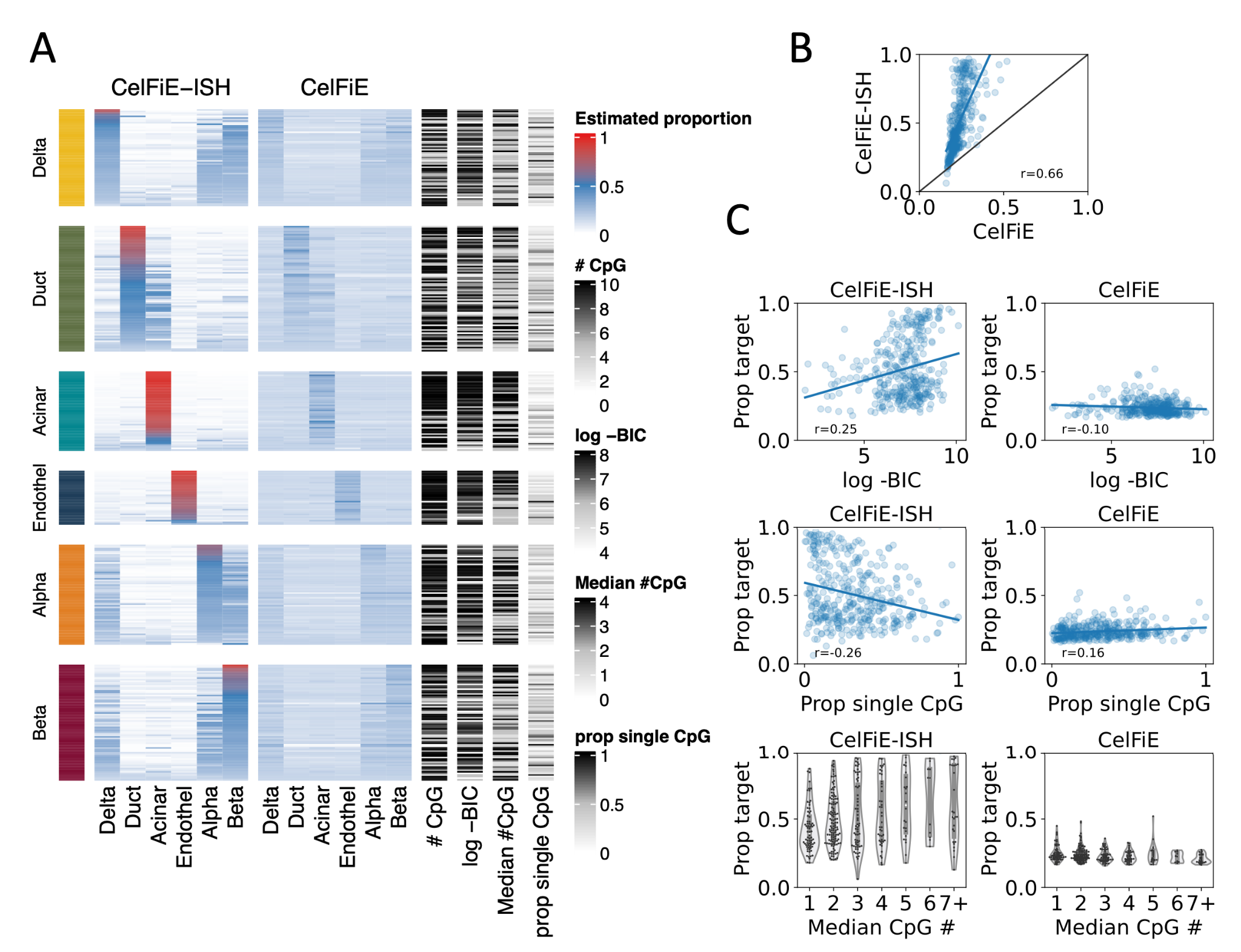
**

### **Figure S11: Differential marker contributions to deconvolution accuracy using TIM regions.** A) Performance of individual TIM markers in identifying the cell-type specificity between 6 pancreatic cell types. Each group on the horizontal axis shows the accuracy of identifying reads from a specific cell type (i.e. the “yellow” section is performance only on reads derived the pancreatic Delta cell holdout sample). Within each group all TIM markers are displayed as rows, and the percentage of reads identified as each of the 6 cell types in a 6-cell type deconvolution is shown as a heatmap. Rows are ordered by accuracy, i.e. markers that assign the highest fraction of reads to the target cell type are at the top. Additional features of each marker are shown at the right: # CpG: number of CpG sites in the region, log -BIC: log of the Bayesian information criterion, Median # CpG: the median number of CpGs per read, prop single CpG: proportion of reads with a single CpG out of all reads overlapping the region. B) Correlation of the percentage of reads assigned to the target cell type (“Prop target”) between CelFIE and CelFiE-ISH. C) Correlation of the percentage of reads assigned to the target cell type (“Prop target”) between each model and each individual marker features (left column for CelFIE-ISH, right for CelFiE).


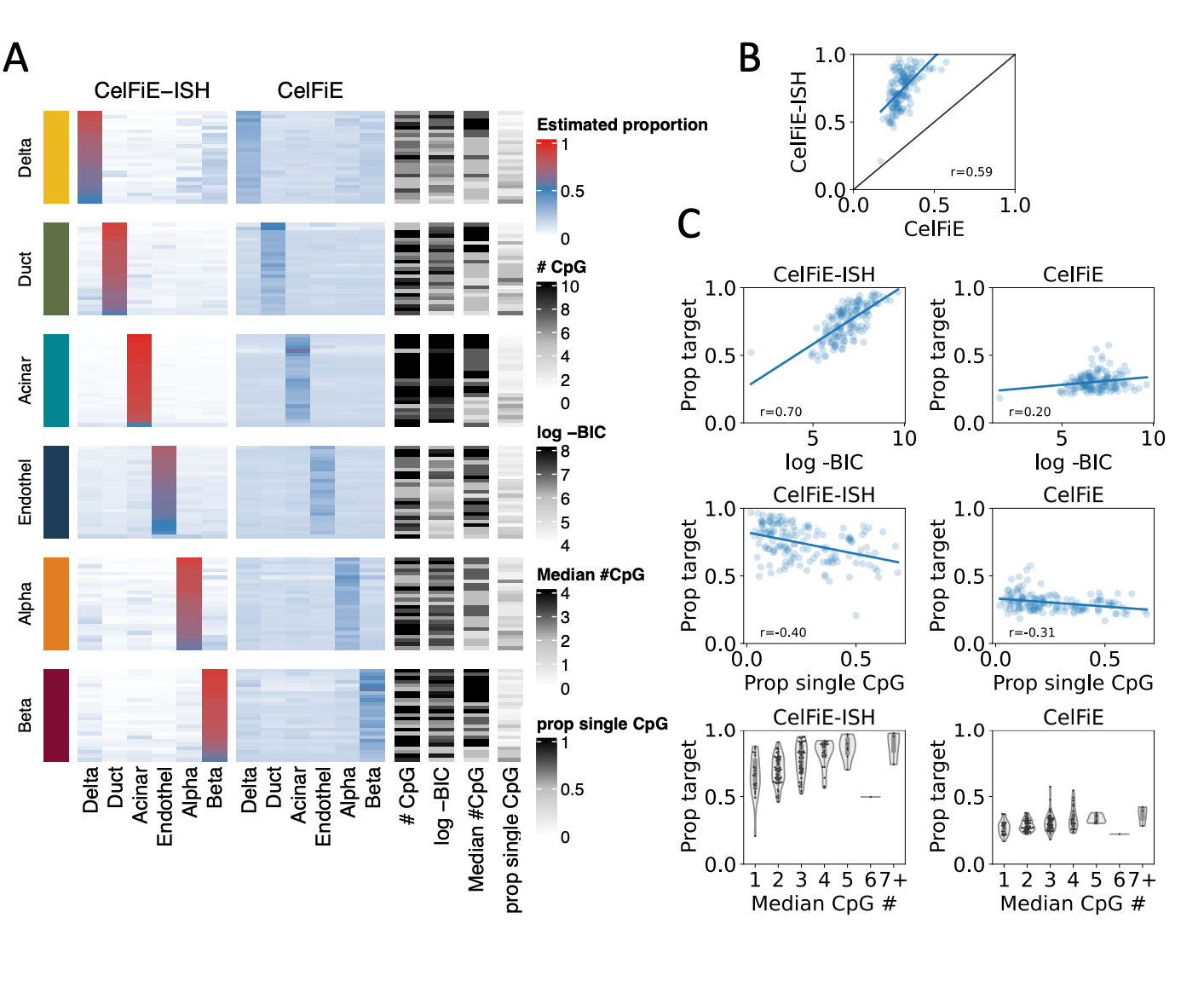


### **Figure S12: Differential marker contributions to deconvolution accuracy using U25 regions.** A) Performance of individual U25 markers in identifying the cell-type specificity between 6 pancreatic cell types. Each group on the horizontal axis shows the accuracy of identifying reads from a specific cell type (i.e. the “yellow” section is performance only on reads derived the pancreatic Delta cell holdout sample). Within each group all U25 markers are displayed as rows, and the percentage of reads identified as each of the 6 cell types in a 6-cell type deconvolution is shown as a heatmap. Rows are ordered by accuracy, i.e. markers that assign the highest fraction of reads to the target cell type are at the top. Additional features of each marker are shown at the right: # CpG: number of CpG sites in the region, log -BIC: log of the Bayesian information criterion, Median # CpG: the median number of CpGs per read, prop single CpG: proportion of reads with a single CpG out of all reads overlapping the region. B) Correlation of the percentage of reads assigned to the target cell type (“Prop target”) between CelFIE and CelFiE-ISH. C) Correlation of the percentage of reads assigned to the target cell type (“Prop target”) between each model and each individual marker features (left column for CelFIE-ISH, right for CelFiE).


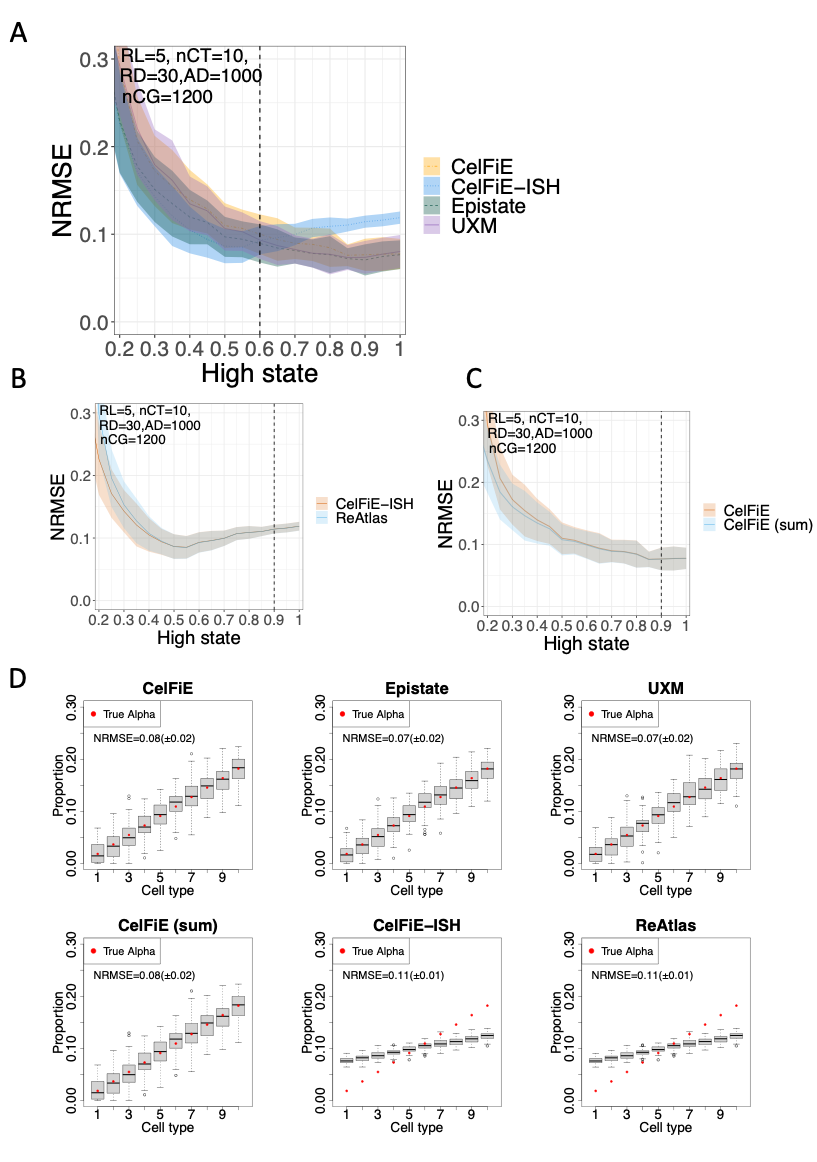


### **Figure S13: shrinkage effect in simulated conditions.** A) NRMSE as a result of varying the [
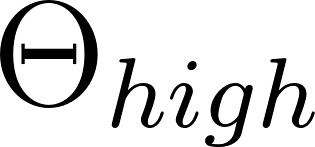
](https://www.codecogs.com/eqnedit.php?latex=%5CTheta_%7Bhigh%7D#0) state from 0.2 to 1.0, when the [
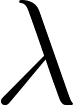
](https://www.codecogs.com/eqnedit.php?latex=%5Clambda#0) is 0.8 (with [
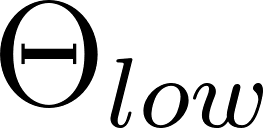
](https://www.codecogs.com/eqnedit.php?latex=%5CTheta_%7Blow%7D#0) [held constant at](https://www.codecogs.com/eqnedit.php?latex=%5CTheta_%7Blow%7D#0) 0.1, using read length RL=5). Shaded area shows standard deviation across 50 replicates.A dotted vertical line shows the condition from panel D below. B) ReAtlas compared to CelFiE-ISH. C) CeFiE-Sum compared to CelFiE. D) Shrinkage at [
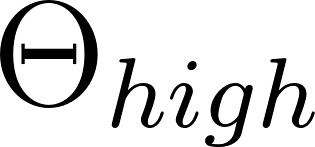
](https://www.codecogs.com/eqnedit.php?latex=%5CTheta_%7Bhigh%7D#0)=0.9.
