# Supplementary material for: CelFiE-ISH: a probabilistic model for multi-cell type deconvolution from single-molecule DNA methylation haplotypes
Source: Genome Biol. 2024 Jun 10;25:151. doi: 10.1186/s13059-024-03275-x (PMC11163775; doi:10.1186/s13059-024-03275-x)
Supplement: Supplementary file 4 — Additional file 4. Supplementary methods. [file 13059_2024_3275_MOESM4_ESM.pdf]

The CelFiE-ISH Model

1 Reference Atlas

The reference atlas consists of one matrix $\beta_{t,m}$, with the probability of methylation for cell type t at position m. In this model we do not re-estimate the atlas at each iteration.

2 Mixture

The mixture is one matrix X, with dimensions C reads over M CpG sites.

3 Likelihood

The observed data likelihood is:

$$P(x|\alpha,\beta) = \prod_c \sum_t \alpha_t P(x_c|\beta_t) = \\ \prod_c \sum_t \alpha_t \prod_m \beta_{t,m}^{x_{c,m}} (1-\beta_{t,m})^{1-x_{c,m}} \tag{1}$$

The observed data log-likelihood is:

$$logP(x|\alpha,\beta) = \sum_c log(\sum_t \alpha_t \prod_m \beta_{t,m}^{x_{c,m}} (1-\beta_{t,m})^{1-x_{c,m}}) = \\ \sum_c logsumexp\left\{ log(\alpha_t \prod_m \beta_{t,m}^{x_{c,m}} (1-\beta_{t,m})^{1-x_{c,m}}) \right\} = \\ \sum_c logsumexp\left\{ log(\alpha_t) + \sum_m x_{c,m} log(\beta_{t,m}) + (1-x_{c,m}) log(1-\beta_{t,m}) \right\} \tag{2}$$

The complete data likelihood is:

$$P(x,z|\alpha,\beta) = P(x|z,\beta)P(z|\alpha)$$

(3)

Where the first term is

$$log(P(x|z,\beta)) = \sum_{t,c,m} log\left[\beta_{t,m}^{z_{t,c}x_{c,m}}(1-\beta_{t,m})^{z_{t,c}(1-x_{c,m})}\right[\right]$$
$$= \sum_{t,c,m} z_{t,c}[x_{c,m}log(\beta_{t,m}) + (1-x_{c,m})log(1-\beta_{t,m})]$$

(4)

and the second term is

$$log(P(z|\alpha)) = \sum_{t,c} log(\alpha_t^{z_{t,c}}) = \sum_{t,c} z_{t,c}log(\alpha_t)$$

(5)

4 Q function

As z in unknown, we define $\tilde{p}$ as the probability of z:

$$P(z_{t,c} = 1|\alpha,\beta) =: \tilde{p}_{t,c}$$

Q is the expected value of the log-likelihood function.

At iteration i, the Q-function is:

$$Q_i = \mathbb{E}_{z|x,\alpha^i,\beta}(logP(x,z|\alpha,\beta)) =$$
$$\sum_{t,c}\tilde{p}_{t,c}^i \sum_m [x_{c,m}log(\beta_{t,m}) + (1-x_{c,m})log(1-\beta_{t,m})] +$$
$$\sum_{t,c}\tilde{p}_{t,c}^i log(\alpha_t)$$

(6)

5 E-step

In the E-step we estimate the latent variable z and use it to define the Q function.

$$P(z_{t,c} = 1 | x_c, \beta, \alpha) = \frac{\alpha_t \prod_m \beta_{t,m}^{x_{m,c}} (1 - \beta_{t,m})^{1 - x_{m,c}}}{\sum_k \alpha_k \prod_m \beta_{k,m}^{x_{m,c}} (1 - \beta_{k,m})^{1 - x_{m,c}}} =: \tilde{p}_{t,c} \tag{7}$$

6 M-step

In the M-step we maximize the Q function, holding the estimate for the latent variable z constant and maximizing α.

$$\alpha_t = \frac{\sum_c \tilde{p}_{t,c}}{C}$$

The CelFiE-ISH ReAtlas Model

7 Reference Atlas

The reference atlas consists of two matrices, $Y_{t,m}$ and $D^Y_{t,m}$, with the number of methylated and total reads for cell type t at position m respectively. We assume $Y_{t,m}$ is drawn from a Binomial distribution with $\beta_{t,m}$ being the true methylation probability and $D^Y_{t,m}$ being the number of trials. We re-estimate the atlas at each iteration.

8 Mixture

The mixture is one matrix X, with dimensions C reads over M CpG sites.

9 Likelihood

The observed data likelihood is:

$$P(x|\alpha, \beta) = P(x|\alpha, \beta) P(Y|\beta) = \prod_c \sum_t \alpha_t P(x_c|\beta_t) P(Y|\beta) =$$

$$\prod_c \left\{ \sum_t \alpha_t \prod_m \beta_{t,m}^{x_{c,m}} (1 - \beta_{t,m})^{1 - x_{c,m}} \right\} \prod_t \prod_m \left\{ \beta_{t,m}^{Y_{t,m}} (1 - \beta_{t,m})^{D^{Y_{t,m}} - Y_{t,m}} \right\} \tag{8}$$

The observed data log-likelihood is:

$$logP(x|\alpha,\beta) = \sum_c log(\sum_t \alpha_t \prod_m \beta_{t,m}^{x_{c,m}}(1-\beta_{t,m})^{1-x_{c,m}}) + log(P(Y|\beta)) =$$

$$\sum_c logsumexp\left\{ log(\alpha_t \prod_m \beta_{t,m}^{x_{c,m}}(1-\beta_{t,m})^{1-x_{c,m}}) \right\} + log(P(Y|\beta)) =$$

$$\sum_c logsumexp\left\{ log(\alpha_t) + \sum_m x_{c,m} log(\beta_{t,m}) + (1-x_{c,m}) log(1-\beta_{t,m}) \right\} + log(P(Y|\beta)) =$$

$$\sum_c logsumexp\left\{ log(\alpha_t) + \sum_m x_{c,m} log(\beta_{t,m}) + (1-x_{c,m}) log(1-\beta_{t,m}) \right\} +$$

$$\sum_{t,m}\left\{ Y_{t,m} log\beta_{t,m} + (D^{Y_{t,m}} - Y_{t,m}) log(1-\beta_{t,m}) \right\}$$

(9)

The complete data likelihood is:

$$P(x,z,Y|\alpha,\beta) = P(x|z,\beta)P(z|\alpha)P(Y|\beta)$$

(10)

The first term is

$$log(P(x|z,\beta)) = \sum_{t,c,m} log\left[\beta_{t,m}^{z_{t,c}x_{c,m}}(1-\beta_{t,m})^{z_{t,c}(1-x_{c,m})}[\right]$$

$$= \sum_{t,c,m} z_{t,c}[x_{c,m} log(\beta_{t,m}) + (1-x_{c,m}) log(1-\beta_{t,m})]$$

(11)

The second term is

$$log(P(z|\alpha)) = \sum_{t,c} log(\alpha_t^{z_{t,c}}) = \sum_{t,c} z_{t,c} log(\alpha_t)$$

(12)

The third term is

$$log(P(Y|\beta)) = \sum_{t,m} Y_{t,m} log\beta_{t,m} + (D^{Y_{t,m}} - Y_{t,m}) log(1 - \beta_{t,m})$$

(13)

10 Q function

As z in unknown, we define $\tilde{p}$ as the probability of z:

$$P(z_{t,c} = 1|\alpha, \beta) =: \tilde{p}_{t,c}$$

Q is the expected value of the log-likelihood function.

At iteration i, the Q-function is:

$$Q_i = \mathbb{E}_{z|x,\alpha^i,\beta^i}(logP(x, z, Y|\alpha, \beta)) =$$
$$\sum_{t,c} \tilde{p}^i_{t,c} \sum_m [x_{c,m} log(\beta_{t,m}) + (1 - x_{c,m}) log(1 - \beta_{t,m})] +$$
$$\sum_{t,c} \tilde{p}^i_{t,c} log(\alpha_t) +$$
$$\sum_{t,m} Y_{t,m} log\beta_{t,m} + (D^{Y_{t,m}} - Y_{t,m}) log(1 - \beta_{t,m})$$

(14)

11 E-step

In the E-step we estimate the latent variable z and use it to define the Q function.

$$P(z_{t,c} = 1|x_c, \beta, \alpha) = \frac{\alpha_t \prod_m \beta_{t,m}^{x_{m,c}} (1 - \beta_{t,m})^{1-x_{m,c}}}{\sum_k \alpha_k \prod_m \beta_{k,m}^{x_{m,c}} (1 - \beta_{k,m})^{1-x_{m,c}}} =: \tilde{p}_{t,c}$$

(15)

12 M-step

In the M-step we maximize the Q function, holding the estimate for the latent variable z constant and maximizing α.

$$\alpha_t = \frac{\sum_c \tilde{p}_{t,c}}{C}$$

Next, we re-estimate the atlas:

$$\beta_{t,m} = \frac{Y_{t,m} + \sum_c \tilde{p}_{t,c} x_{c,m}}{D^{Y_{t,m}} + \sum_c \tilde{p}_{t,c}}$$

(16)

The Epistate Model

At every marker region, reads are drawn from one of two possible epistates: θ_{high} and θ_{low}. Each epistate consists of a set of binomial distributions $\theta = \{\theta_1, \theta_2, ..., \theta_m\}$, one per CpG site covered by the marker region. θ_{high} is arbitrarily defined to be the epistate with higher mean methylation. Cell types differ by the probability of observing each epistate in each region.

13 Reference Atlas

The reference atlas consists of one matrix $\lambda_{t,c}$, with the probability of observing θ_{high} under cell type t at read c. Within a genomic region λ does not vary between reads, leaving λ_t. Additionally, for every position we know $\theta_{high,m}$ and $\theta_{low,m}$ (see below). The overall probability of methylation per position is:

$$\beta_{t,m} = \lambda_t \theta_{high,m} + (1 - \lambda_t)\theta_{low,m}$$

14 Mixture

The mixture is one matrix X, with dimensions C reads over M CpG sites.

15 Likelihood

The observed data likelihood is:

$$P(x|\alpha, \theta_{high}, \theta_{low}, \lambda) = \prod_c \sum_t \alpha_t \left\{ \lambda_{t,c} \prod_m \left[\theta_{high}^{x_{c,m}} (1 - \theta_{high})^{1 - x_{c,m}} \right] + (1 - \lambda_{t,c}) \prod_m \left[\theta_{low}^{x_{c,m}} (1 - \theta_{low})^{1 - x_{c,m}} \right] \right\}$$

(17)

The observed data log-likelihood is:

$$logP(x|\alpha, \theta_{high}, \theta_{low}, \lambda) = \sum_c log(\sum_t \alpha_t \Bigg\{ \lambda_{t,c} \prod_m \Big[\theta_{high}^{x_{c,m}} (1-\theta_{high})^{1-x_{c,m}} \Big] +$$
$$(1-\lambda_{t,c}) \prod_m \Big[\theta_{low}^{x_{c,m}} (1-\theta_{low})^{1-x_{c,m}} \Big] \Bigg\}) =$$
$$\sum_c logsumexp_t \Bigg\{ log(\alpha_t) + log(\lambda_{t,c} \prod_m \Big[\theta_{high}^{x_{c,m}} (1-\theta_{high})^{1-x_{c,m}} \Big] +$$
$$(1-\lambda_{t,c}) \prod_m \Big[\theta_{low}^{x_{c,m}} (1-\theta_{low})^{1-x_{c,m}} \Big]) \Bigg\} =$$
$$\sum_c logsumexp_t \Bigg\{ log(\alpha_t) + logsumexp \Bigg\{ log(\lambda_{t,c}) + \sum_m \Big[x_{c,m} log(\theta_{high}) + (1-x_{c,m}) log(1-\theta_{high}) \Big],$$
$$log(1-\lambda_{t,c}) + \sum_m \Big[x_{c,m} log(\theta_{low}) + (1-x_{c,m}) log(1-\theta_{low}) \Big] \Bigg\} \Bigg\}$$

(18)

z is the indicator for α and μ is the indicator for λ. The complete data likelihood is:

$$P(x, z, \mu|\alpha, \theta_{high}, \theta_{low}, \lambda) = P(x|\mu, \theta_{high}, \theta_{low}) P(z|\alpha) P(\mu|z, \lambda)$$

(19)

The first term is

$$log(P(x|\mu, \theta_{high}, \theta_{low})) = log(\prod_c \prod_m \Big[\theta_{high,m}^{\mu_c x_{c,m}} (1-\theta_{high,m})^{\mu_c(1-x_{c,m})}$$
$$\theta_{low,m}^{(1-\mu_c)x_{c,m}} (1-\theta_{low,m})^{(1-\mu_c)(1-x_{c,m})} \Big]) =$$
$$\sum_{c,m} \Big[\mu_c x_{c,m} log(\theta_{high,m}) + \mu_c (1-x_{c,m}) log(1-\theta_{high,m}) +$$
$$(1-\mu_c) x_{c,m} log(\theta_{low,m}) + (1-\mu_c)(1-x_{c,m}) log(1-\theta_{low,m}) \Big]$$

(20)

The second term is

$$log(P(z|\alpha)) = \sum_{t,c} log(\alpha_t^{z_{t,c}}) = \sum_{t,c} z_{t,c} log(\alpha_t) \tag{21}$$

The third term is

$$\begin{aligned} log(P(\mu|z,\lambda)) = log(\prod_t \prod_c \lambda_{t,c}^{z_{t,c}\mu_c}(1-\lambda_{t,c})^{z_{t,c}(1-\mu_c)}) = \\ \sum_{t,c} \Big[z_{t,c}\mu_c log(\lambda_{t,c}) + z_{t,c}(1-\mu_c) log(1-\lambda_{t,c}) \Big] \end{aligned} \tag{22}$$

16 Q function

As z in unknown, we define $\tilde{p}$ as the posterior probability of z:

$$P(z_{t,c} = 1|\alpha, x) =: \tilde{p}_{t,c}$$

Similarly,

$$P(\mu_c = 1|z, x) =: \tilde{q}_c$$

Note that λ, θ_{high}, θ_{low} and by extension β are always given and not re-estimated. For simplicity, we left them out of the conditional statements.
Q is the expected value of the log-likelihood function.

At iteration i, the Q-function is:

$$\begin{aligned} Q_i = \mathbb{E}_{z,\mu|x,\alpha^i,\lambda,\theta_{high},\theta_{low}}(logP(x,z,\mu|\alpha^i,\theta_{high},\theta_{low},\lambda)) = \\ \sum_{t,c} \Big\{ \tilde{p}_{t,c}\tilde{q}_c \sum_m \Big[x_{c,m} log(\theta_{high,m}) + (1-x_{c,m}) log(1-\theta_{high,m}) \Big] + \\ \tilde{p}_{t,c}(1-\tilde{q}_c) \sum_m \Big[x_{c,m} log(\theta_{low,m}) + (1-x_{c,m}) log(1-\theta_{low,m}) \Big] \Big\} \\ \sum_{t,c} \Big\{ \tilde{p}_{t,c} log(\alpha_t^i) \Big\} + \\ \sum_{t,c} \Big\{ \tilde{p}_{t,c}\tilde{q}_c log(\lambda_{t,c}) + \tilde{p}_{t,c}(1-\tilde{q}_c) log(1-\lambda_{t,c}) \Big\} \end{aligned} \tag{23}$$

17 E-step

In the E-step we estimate the latent variables z and μ and use them to define the Q function.

$$P(\mu_c = 1|x, \alpha) = \sum_t P(z_{t,c} = 1|x, \alpha_t)P(\mu_c = 1|z_{t,c} = 1, x, \alpha) =$$
$$\sum_t \tilde{p}_{t,c}P(\mu_c = 1|z_{t,c} = 1, x) \propto \sum_t \tilde{p}_{t,c}P(x|\mu_c = 1, z_{t,c} = 1)P(\mu_c = 1|z_{t,c} = 1) =$$
$$\sum_t \tilde{p}_{t,c}P(x|\mu_c = 1)P(\mu_c = 1|z_{t,c} = 1) = \sum_t \tilde{p}_{t,c}\lambda_t P(x|\mu_c = 1) =$$
$$\sum_t \tilde{p}_{t,c}\lambda_t \prod_m \theta_{high}^{x_{c,m}}(1 - \theta_{high})^{1-x_{c,m}} \tag{24}$$

Since μ can only take on two values, we constrain

$$P(\mu_c = 1|x, \tilde{p}) + P(\mu_c = 0|x, \tilde{p}) = 1$$

As above:

$$P(\mu_c = 0|x, \tilde{p}) = \sum_t \tilde{p}_{t,c}(1 - \lambda_t) \prod_m \theta_{low}^{x_{c,m}}(1 - \theta_{low})^{1-x_{c,m}}$$

Finally:

$$P(\mu_c = 1|x, \alpha) = \frac{\sum_t \tilde{p}_{t,c}\lambda_t \prod_m \theta_{high}^{x_{c,m}}(1 - \theta_{high})^{1-x_{c,m}}}{\sum_t \tilde{p}_{t,c}\lambda_t \prod_m \theta_{high}^{x_{c,m}}(1 - \theta_{high})^{1-x_{c,m}} + \sum_t \tilde{p}_{t,c}(1 - \lambda_t) \prod_m \theta_{low}^{x_{c,m}}(1 - \theta_{low})^{1-x_{c,m}}} \tag{25}$$

We do the same for z:

$$P(z_{t,c} = 1|x, \alpha_t) \propto P(x|z_{t,c} = 1, \alpha_t)P(z_{t,c} = 1|\alpha_t) = \Big[\lambda_{t,c}P(x|\mu_c = 1) + (1 - \lambda_{t,c})P(x|\mu_c = 0)\Big]\alpha_t$$
$$= \alpha_t\lambda_{t,c} \prod_m \Big[\theta_{high}^{x_{c,m}}(1 - \theta_{high})^{1-x_{c,m}}\Big] + \alpha_t(1 - \lambda_{t,c}) \prod_m \Big[\theta_{low}^{x_{c,m}}(1 - \theta_{low})^{1-x_{c,m}}\Big] \tag{26}$$

Then normalize so that every read comes from a cell type.

18 M-step

In the M-step we maximize the Q function, holding the estimate for the latent variables constant and maximizing α. The only term in the Q function with α is identical to CelFiE and CelFiE+, so the maximization step is the same.

$$\alpha_t = \frac{\sum_c \tilde{p}_{t,c}}{C}$$

Estimating Epistates in the Reference Atlas

For each marker region in the Epistate reference, we estimate Θ_{high}, Θ_{low} and λ_t. First, we jointly examine all reads from the entire reference dataset. We assume each read is associated with either Θ_{high} or Θ_{low}. υ_j is the prior probability for epistate $j \in [1, 2]$. At the expectation step, we update the posterior probability of each read $P_{j,c}$ given Θ. At the maximization step, we estimate the hidden state Θ, and υ_j.

19 Likelihood

The observed data likelihood is:

$$P(x|\Theta_{high}, \Theta_{low}, \upsilon) = \prod_c \sum_{j=1}^{2} \upsilon_j \left[\prod_m \theta_j^{x_{c,m}} (1-\theta_j)^{1-x_{c,m}} \right]$$

Expectation

$$P_{j,c} = \frac{\upsilon_j \prod_m \theta_{m,j}^{x_{c,m}} (1-\theta_{k,j})^{1-x_{c,m}}}{\sum_{j=1}^{2} \upsilon_j \prod_m \theta_{m,j}^{x_{c,m}} (1-\theta_{k,j})^{1-x_{c,m}}}$$

Maximization

$$\theta_{m1} = \frac{pseudocount + \sum_c P_{1,c} x_{c,m}}{2 * pseudocount + \sum_c P_{1,c}}$$

$$\upsilon_1 = \frac{pseudocount + \sum_c P_{1,c}}{2 * pseudocount + C}$$

Then, we split the reference by cell type. For each cell type, λ if the probability of observing Θ_{high}. For each subset:

$$\lambda_t = \frac{\sum_c P_{1,c}}{C}$$

Worst possible RMSE

Let $Y = [Y_1, Y_2, \ldots, Y_n]$ be a vector of true cell type fractions in a mixture, ordered from smallest to largest $Y_1 \leq Y_2 \leq \ldots \leq Y_n$ and $\hat{Y} = [\hat{Y}_1, \hat{Y}_2, \ldots, \hat{Y}_n]$ be the estimated values. The RMSE is defined as

$$\sqrt{\frac{1}{n} \sum_{i=1}^{n} (\hat{Y}_i - Y_i)^2}$$

As these are fractions we can add the constraint that $\sum_{i=1}^{n} Y_i = 1$ and $0 \leq Y_i \leq 1$ for all i. This is also true for the estimates: $\sum_{i=1}^{n} \hat{Y}_i = 1$ and $0 \leq \hat{Y}_i \leq 1$ for all i.

For the worst-case estimation, i.e. the largest RMSE, let $\hat{Y}_1 = 1$ and $\hat{Y}_i = 0$ for $i \neq 1$. The squared error terms are then $(1 - Y_1)^2$ for $i = 1$ and Y_i^2 for $i \neq 1$.

To prove this results in the maximum RMSE, consider any other estimate $\hat{Y}'$. This implies, for some $j \neq 1$, $\hat{Y}_j' > 0$.

The squared error term would then be $(1 - Y_1 - \hat{Y}_j')^2$ for $i = 1$, $(\hat{Y}_j' - Y_j)^2$ for $i = j$, and Y_i^2 for $i \neq 1, j$. Since $\hat{Y}_j'$ is non-negative and ≤ 1, $(1 - Y_1 - \hat{Y}_j')^2 < (1 - Y_1)^2$ and $(\hat{Y}_j' - Y_j)^2 < {Y_j}^2$.

The entire expression is therefore smaller than the worst-case estimation. Intuitively, since Y_1 is the smallest, its error term has the largest impact on increasing the RMSE when estimated far from its true value. Thus, any other estimation would result in a lower RMSE.

WGBS Data Processing

In order to convert BAM files to the Biscuit epiread format, we first generated a SNP file from the VCF files requiring $GQ \geq 15$ for positions overlapping a dbSNP common allele, and requiring $GQ \geq 60$ for all other positions. DbSNP common allele table was downloaded from UCSC for the hg19 assembly, and was processed with:
https://github.com/ekushele/methylseq/blob/master/bin/processUcscDbsnp.pl.

From the processed file, we included only 'snv' records. The formatted-snv file was zipped and indexed with the `tabix -s 1 -b 2 -e 3` command. This file was passed to bcftools annotate (v1.9) to annotate the header of VCF files:
`bcftools annotate WHITELIST -O z -a {COMMON_DBSNP_FILE} -h common_dbsnp.hdr -c CHROM,FROM,TO,TYPE,COMMON_SOME,COMMON_ALL,REF_MIN,ALT_MIN,REF_DBSNP, ALT_DBSNP,REF_ALL,ALT_ALL,RSID,MAX_MAF {VCF_FILE}`.
(common_dbsnp.hdr can be found at:
https://github.com/ekushele/methylseq/blob/master/assets/common_dbsnp.hdr).

The redhead file was indexed with `tabix -p vcf`. From the re-headed files, we included variants with $GQ \geq 60$ for heterozygous variants for positions not overlapping the `COMMON_DBSNP_FILE` with `bcftools view -O z -i 'ALT!="N" & ALT!="." & ((COUNT(GT=="0/1")` $\geq 1\&COMMON\_ALL == 1\&MAX\_MAF \geq 0.05) \,|\, (COUNT(GT == "0/1"\&GQ \geq 60) \geq 1))'\{REHEAD\_VCF\} > \{DBSNP\_HET60\}$.

`{DBSNP_HET60}` was indexed with `tabix -p vcf`. For all other variants, we excluded variants below 10 and parsed the file to be in bed format with the following command:
`bcftools query -u -i 'GT="0/1" & GQ` $\geq 10' - -format'$

$\%CHROM\%POS\%POS\%REF\%ALT[\%GT\%GQ\%SP\%AC\%AF1]\%RSID\%$
$COMMON\_ALL\%MAX\_MAF\%REF\_MIN\%ALT\_MIN'\{DBSNP\_HET60\}|$
$awk - vOFS = "nt"'\{\$2 = \$2 - 1; print\}' > \{SNP\_FILE\}$.
Then, blacklist regions were excluded from BAM files with the command `bedtools intersect`(v2.29.1) using the BAM and a whitelist as input files, and additional command line arguments`'-ubam -f 1.0'`.

Epiread files were produced with the `biscuit epiread` command for whitelist-BAM files where a SNP file was given as input to the `-B` argument: `'-B SNP_FILE'`. The epiread files were sorted by names using the command `'-k2,2 -k1,1 -k4,4 -k3,3n'` , and they were converted to a bed-like format, merging paired-end epiread records together using the script available at
`https://github.com/ekushele/methylseq/blob/master/bin/epiread_pairedEnd_convertion`
in debug mode.

The CpG file was downloaded from the Biscuit QC assets release page:
`https://github.com/huishenlab/biscuit/releases`
These merged files were sorted by position using the command `sort -k1,1Vf -k 2,2n -k 3,3n` and then tabixed using the `'tabix -0 -p bed'` command. The original epireads (before merging) were sorted with `sort -k1,1Vf -k5,5V` and tabixed with `tabix -0 -s 1 -b 5 -e 5`.
