# Supplementary material for: CelFiE-ISH: a probabilistic model for multi-cell type deconvolution from single-molecule DNA methylation haplotypes
Source: Genome Biol. 2024 Jun 10;25:151. doi: 10.1186/s13059-024-03275-x (PMC11163775; doi:10.1186/s13059-024-03275-x)
Supplement: Supplementary file 5 — Additional file 5. Review history. [file 13059_2024_3275_MOESM5_ESM.docx]

Review History

**First round of review**

**Reviewer 1**

**Were you able to assess all statistics in the manuscript, including the appropriateness of statistical tests used?**

Yes, and I have assessed the statistics in my report.

**Comments to author:**

The manuscript by Unterman et al. titled "Multi-cell type deconvolution using a probabilistic model for single-molecule DNA methylation haplotypes" presents a novel software tool aimed at improving the deconvolution of mixed cell types from tissue or blood samples, based on DNA methylation haplotypes at the single-molecule level. This method addresses a significant gap in genomic profiling, as current techniques can profile multiple individual CpG positions, but few models exploit these single-molecule methylation haplotypes for cell type deconvolution. The study's authors developed two new models: an adaptation of an existing model, CelFiE, to incorporate methylation haplotype information (named CelFiE Integrated Single-molecule Haplotypes or CelFiE-ISH), and a second approach that extends previous 2-cell type methods (Epistate) to account for multiple cell types (CelFiE-ReAtlas). The performance of these models was compared against existing deconvolution tools using simulated whole-genome methylation data, in silico mixtures of real data, and a range of different cell types and conditions.

Evaluation of the Software:

Utility and Ease of Use:

Broad Utility: The software seems to have broad utility, especially for analyzing complex DNA methylation data to deconvolute mixed cell types. This is crucial in various biological and clinical settings, such as cancer research, where understanding cell composition in tissue samples is vital.

Ease of Installation and Use: The software installation was easy with some vignettes/examples although it does not present a step by step process which may limit the adoption of the method. I had some issues running the larger examples that preclude a more complete evaluation of the library. It is vital that the process can be reproduced in different systems. Some warnings for epiparser appeared in both an Ubuntu and a RedHat Unix environments, when trying to run the larger deconvolution examples.

Advancement Over Previous Tools:

The manuscript demonstrates that the new models, particularly CelFiE-ISH, significantly improve deconvolution accuracy (by ~30%) over existing tools. This includes better detection of rare ell types and improved accuracy in different simulation scenarios. These improvements suggest a significant advance over previously published tools.

Novel Features:

The key novel feature is the integration of methylation haplotype information into the deconvolution process. This allows for more accurate and detailed analysis of cell types based on DNA methylation patterns, especially in detecting rare cell types.

Appropriateness and Description of Methods:

The methods seem appropriate for the study's aims, leveraging complex statistical models and simulations to test the efficacy of the new software. The manuscript provides a detailed description of these methods, allowing for replication and comparison with other analyses.

Support for Conclusions:

The conclusions drawn in the study are adequately supported by the data shown, particularly the improved performance of the new models in various test conditions and simulations.

Replication and Comparison Details:

Sufficient details are provided for replication, and the manuscript includes comparisons with related analyses, enhancing the study's credibility.

Interest to the Field and Broader Audience:

The paper is of broad interest to researchers in fields like genomics, epigenetics, and bioinformatics. It may also attract a wider audience of biologists and clinicians interested in advanced methods for analyzing complex genomic data.

Comments:

Figure 1B: The normalized RMSE need some additional explanation of the concept in this context is this assuming that the cell types are unevenly distributed in the mixture or is for the simulation, please expand this in the text.

Figure 1B: In general, it is shown an increase in the error rate with the increase in the number of cells being deconvolved, but the errors are very different across different bins, being relatively stable with less than 5 cells and increasing drastically with more than 10 cell types. Could you please expand on these concepts and areas? Is this error acceptable for the two state models

Figure 1C: why is UXM performance deteriorating with read length?

Figure 2 A: the Θ has drifted on the pdf for the 0.25 and 0.45.

Figure 2 B and C: Again this requires more context about the biology that can or cannot be explained depending on the NRMSE 4% or 15% can be low or high for specific cell types based on the cell with poorest performance. Some explanation is important in this context.

Page 11, lines 36-51: This part of the method is unclear. What is the space of the TIM 100 vs using the top 25 or top 250 markers per cell-type?

Page 11, lines 30-31: The Epistate bias needs some expansion, why is the overestimation happening?

Figure 3B: Please expand on these findings why the instability of a smaller space, again the error needs some context to interpret the error rate observed and the impact on the biological interpretation of the results.

Figure 4E: I do not see a caption for this area of the figure, this needs to be explained better in the caption.

For broader adoption, it is important to provide a step by step process for the installation and some troubleshooting.

**Reviewer 2**

**Were you able to assess all statistics in the manuscript, including the appropriateness of statistical tests used?**

Yes, and I have assessed the statistics in my report.

**Comments to author:**

In this manuscript, the authors reported an improved strategy for methylation-based deconvolution, and detecting rare cell types. An underlying key concept is that, the co-methylation status of multiple adjacent CpG sites on a single DNA molecules, which can be determined from bisulfite sequencing reads, is more informative comparing with the methylation status of single CpG sites, and that can be harnessed for more sensitive deconvolution. While that concept has been utilized in a few previous published studies, the authors demonstrated a significant improvements in terms of accuracy and limit of detection over previous works. They achieved this by benchmarking three existing algorithms, and further adapting one algorithm CelFiE to methylation haplotypes explicitly. They used a recently published set of WGBS data from different cell types to create simulated data with known composition, in order to assess the performance of different algorithms or variants. They showed a clear advantage of CelFiE-ISH over other options, documented the effects on different marker/parameter choices, and identified specific contexts that certain algorithm worked better or worse. Finally, they further provide a proof-of-concept demonstration on another recently published data set from COVID-19 patients. Overall, this is a timely study on how to use methylation for deconvolution and rare cell detection. While the authors did not develop a standalone novel method from the scratch, they did identified CelFiE-ISH as the winning option, and demonstrated a performance better than other published results that I'm aware of. Through simulations and careful assessments, they identified factors that can improve the performance. These insights are highly valuable to the community. I think this manuscript is appropriate for the readers of Genome Biology.

I do not have any major concerns. The simulations were well designed, and the benchmarking is quite rigorous. I have only one comment/suggestion: all the simulations were done based on WGBS data, and I don't have a big problem with that. However, in real world applications, such as the assays developed by Grail and Guardant Health, targeted bisulfite sequencing was used to increase the coverage of the informative markers and sensitivity under certain cost limits. Is it possible to add a simulation on targeted sequencing data, to find out how low they can go in terms of the limit of detection?

**Authors Response**

**Point-by-point responses to the reviewers’ comments:**

Key: Reviewer questions are marked in **bold**. New text added to manuscript in red.

# Reviewer 1

**Figure 1B: The normalized RMSE need some additional explanation of the concept in this context is this assuming that the cell types are unevenly distributed in the mixture or is for the simulation, please expand this in the text.**

*Response: The root mean square error (RMSE) gives us a single metric to measure the fit of the predicted proportions of each model to the ground-truth proportions. This metric takes into account the absolute differences, meaning estimating a common cell type at 33% rather than 30% is equivalent to estimating a rare cell type at 4% rather than 1%. The best possible RMSE is always 0, but the worst possible RMSE depends on the ground truth levels. Considering a mixture of two cell types, if the ground truth is [0, 1], the worst possible RMSE is 1 (if the estimates are [1, 0]). If, however, the ground truth is [0.5, 0.5], the worst possible estimate [1, 0] would only yield an RMSE of 0.5. We calculate the worst possible RMSE individually for every simulated condition and every in silico mixture, and scale this to have a value of 1 in the NRMSE. Normalizing RMSE to the worst possible error allows us to compare errors across conditions that have very different cell type distributions. This is used for simulated data as well as in silico mixtures. The worst possible outcome is always to give an estimate of 1 to the cell type with the lowest ground truth proportion, and an estimate of 0 for all others.*

*We added this explanation in the main text:*

*“We simulated 25 cell types with linearly increasing true proportions, and ran this on each model with varying read lengths (Figure 1B). For each simulation, we calculated a Normalized Root Mean Square Error (NRMSE) between the true cell type proportion vector and the estimated vector. NRMSE is a rescaled version of Root Mean Squared Error (RMSE), where the minimum value is 0 when the estimates are identical to the true cell type proportions, and the maximum value is 1 assigned to the worst possible RMSE given the true cell type proportions (see Methods). This normalization allows for error comparison across simulations that have different true proportions. (see Methods)”.*

In the methods section, the exact NRMSE formulation used is provided:

Where is the vector of true proportions indexed by is the vector of estimated proportions indexed by is the total number of cell types, and is the lowest true proportion. The full derivation of the worst possible RMSE is available in the Supplementary Note.

,

[,](https://www.codecogs.com/eqnedit.php?latex=i#0)

We also added the full proof of worst RMSE to the supplementary note:

Let be a vector of true cell type fractions in a mixture, ordered from

smallest to largest and be the estimated

values. The RMSE is defined as

As these are fractions we can add the constraint that and for all

. This is also true

for the estimates: and for all.

For the worst-case estimation, i.e. the largest RMSE, let [.](https://www.codecogs.com/eqnedit.php?latex=i%20%5Cneq%201#0)

and

for

The squared error terms are then .

for

and

for

To prove this results in the maximum RMSE, consider any other estimate. This

implies, for some [.](https://www.codecogs.com/eqnedit.php?latex=%5Chat%7BY%7D'_j%20%3E%200#0)

,

The squared error term would then be [,](https://www.codecogs.com/eqnedit.php?latex=i%3Dj#0) and .

for

,

for

for

Since is non-negative and .

[,](https://www.codecogs.com/eqnedit.php?latex=%5Cleq%201#0)

and

*The entire expression is therefore smaller than the worst-case estimation. Intuitively, since* *is the smallest, its error term has the largest impact on increasing the RMSE when estimated far from its true value. Thus, any other estimation would result in a lower RMSE.*

**Figure 1B: In general, it is shown an increase in the error rate with the increase in the number of cells being deconvolved, but the errors are very different across different bins, being relatively stable with less than 5 cells and increasing drastically with more than 10 cell types. Could you please expand on these concepts and areas? Is this error acceptable for the two state models**

*Response: We suspect our description of this plot may have been confusing, because each column in the X axis is one specific cell type, not the number of cell types. For each simulation, we simulated 25 cell types with the following (linearly increasing) proportions:*

*[0.00308,0.00615,0.00923,0.01231,0.01538,0.01846,0.02154,0.02462,0.02769,0.03077*

*,0.03385,0.03692,0.04000,0.04308,0.04615,0.04923,0.05231,0.05538,0.05846,0.06154*

,0.06462,0.06769,0.07077,0.07385,0.07692]

*The least abundant cell type (the left-most column in the plot) is approximately 0.3% (1**

*^31^25) of the mixture and the most abundant^3^125 cell type (the right-most column in the plot) is approximately 7.7% of the mixture (25*). These are chosen to sum to 1 (see below).*

*This ground truth proportion is shown as a red circle for each cell type column, and the boxplot indicates the estimated proportion of this cell type across 50 simulations with the same ground truth. We did not test the effect of the number of simulated cell types on the error. We selected 25 cell types to be consistent with the simulations in the Caggiano paper* [*(Caggiano*](https://paperpile.com/c/71qymx/9WpW) [*et*](https://paperpile.com/c/71qymx/9WpW) [*al.*](https://paperpile.com/c/71qymx/9WpW) [*2021)*](https://paperpile.com/c/71qymx/9WpW) *and in the same ballpark as the number of human cell types available in the Loyfer atlas* [*(Loyfer*](https://paperpile.com/c/71qymx/ViEt) [*et*](https://paperpile.com/c/71qymx/ViEt) [*al.*](https://paperpile.com/c/71qymx/ViEt) [*2023)*](https://paperpile.com/c/71qymx/ViEt)*.*

*The caption for figure 1B was edited to reflect this, and now reads:*

*Deconvolution results for simulated mixtures of 25 cell types, with the left-most cell type having a ground truth proportion of 0.00308 (red circle), and each cell type increasing linearly by 0.00308 until the right-most cell type, which has a proportion of 0.07692 (the increment is set to 0.00308 or 1/325 so they sum to 1, see Methods). The deconvolution estimates for each cell type is shown in a gray box plot: the boxes represent the first to third quartiles of 50 independent simulations, with whiskers extending up to 1.5 times this interquartile range. Outliers are denoted by dots. Each deconvolution model is shown separately, with a read length of 5 (left column) and 1 (right column). The Normalized Root Mean Square Error (NMRSE) and NRMSE standard deviation are shown for each.*

*This was added to the methods section:*

*“The* *we used for 25 simulated cell types was: [0.00308,0.00615,0.00923,0.01231,0.01538,0.01846,0.02154,0.02462,0.02769,0.03077*

*,0.03385,0.03692,0.04000,0.04308,0.04615,0.04923,0.05231,0.05538,0.05846,0.06154*

*,0.06462,0.06769,0.07077,0.07385,0.07692]*

*Or**, with T=25. “*

***Figure 1C: why is UXM performance deteriorating with read length?***

*UXM assigns each read to a class: methylated (M), unmethylated (U) or ambiguous (X). X reads are subsequently discarded. In the n-state simulation model, methylation probabilities are drawn from a random uniform distribution. Therefore, with longer reads, most reads become ambiguous and are uninformative for UXM.*

*We added the following text to elaborate on this:*

*“In the 25 cell type simulation, Epistate and UXM performed poorly (Figure 1B-C). Both of these models assume two methylation states, an assumption which is violated in this simulation where each cell type is defined by a unique methylation pattern. We only investigated UXM with read lengths greater than 4, because the UXM algorithm itself removes reads with less than 4 CpGs. Since UXM requires all CpGs in* *a single fragment to have a consistent methylation state (an assumption violated in this simulation), it performs worse as the read lengths increase.*

**Figure 2 A: the Θ has drifted on the pdf for the 0.25 and 0.45.**

*Response: Thank you, fixed.*

**Figure 2 B and C: Again this requires more context about the biology that can or cannot be explained depending on the NRMSE 4% or 15% can be low or high for specific cell types based on the cell with poorest performance. Some explanation is important in this context.**

*Response: We believe that the question posed here may have stemmed from the confusion we created about how the plot is structured and how NRMSE was calculated (see our response to Figure 1B above, and please indicate whether our updated and improved descriptions there clarify the question posed here.) As discussed above, NRMSE is calculated across all 25 cell types and simply scaled to the maximum possible RMSE for the simulated cell type proportions. In this case, every plot in Figure 2B and 2C has exactly the same simulated cell type proportions, and thus the same exact scaling factor. Please let us know if this requires further clarification.*

*We would like to apologize for incorrectly describing the NRMSE standard deviations as “IQR” and have corrected this in this version. Perhaps that added to the confusion.*

**Page 11, lines 36-51: This part of the method is unclear. What is the space of the TIM 100 vs using the top 25 or top 250 markers per cell-type?**

*Response: First, we attempted to clarify the main text to clarify our general approach, adding the new text in red below:*

***“****Marker selection is an important aspect of reference-based deconvolution, which involves selecting a small set of genomic regions with cell-type specific methylation patterns. Because there is no single agreed upon method for marker selection, we generated and compared the markers using the marker selection methods from the two leading studies* [*(Caggiano*](https://paperpile.com/c/71qymx/9WpW+ViEt) [*et*](https://paperpile.com/c/71qymx/9WpW+ViEt) [*al.*](https://paperpile.com/c/71qymx/9WpW+ViEt) [*2021;*](https://paperpile.com/c/71qymx/9WpW+ViEt) [*Loyfer*](https://paperpile.com/c/71qymx/9WpW+ViEt) [*et*](https://paperpile.com/c/71qymx/9WpW+ViEt) [*al.*](https://paperpile.com/c/71qymx/9WpW+ViEt) [*2023)*](https://paperpile.com/c/71qymx/9WpW+ViEt)*. First, we generated markers using the method provided in the CelFiE package, called Tissue Informative Markers (TIMs)* [*(Caggiano*](https://paperpile.com/c/71qymx/9WpW) [*et*](https://paperpile.com/c/71qymx/9WpW) [*al.*](https://paperpile.com/c/71qymx/9WpW) [*2021)*](https://paperpile.com/c/71qymx/9WpW)*. In the TIM method, CpG sites are selected based on the distance between the percent methylation of a target cell type and the median percent methylation of the CpG across all cell types. Then, a block of +-250bp from the informative CpG is used. We used the default suggested number of TIMs per cell type, 100. As these regions may overlap both within a cell type and between cell types, we merged down the list to non-overlapping genomic intervals. This generated a set of 1665 regions. Next, we imported the cell-type specific regions identified by Loyfer et al.[1]. The first of these included the top 25 cell type specific markers per cell type, which includes 770 regions (the “U25” set). The* *second includes the top 250 markers per cell type, which included 7,475 regions (the “U250” set)* [*(Loyfer*](https://paperpile.com/c/71qymx/ViEt) [*et*](https://paperpile.com/c/71qymx/ViEt) [*al.*](https://paperpile.com/c/71qymx/ViEt) [*2023)*](https://paperpile.com/c/71qymx/ViEt)*. The method used to select the U25 and U250 markers used blocks of 5 or more CpGs, according to the difference between average methylation of the target cell type against all other cell types.”*

*We have also added some relevant background to the Background section:*

*“Most deconvolution approaches use selection criteria to find small genomic regions that best inform cell-type identity. This process reduces computation time and storage requirements* [*(Caggiano*](https://paperpile.com/c/71qymx/9WpW+c88U6) [*et*](https://paperpile.com/c/71qymx/9WpW+c88U6) [*al.*](https://paperpile.com/c/71qymx/9WpW+c88U6) [*2021;*](https://paperpile.com/c/71qymx/9WpW+c88U6) [*Zheng*](https://paperpile.com/c/71qymx/9WpW+c88U6) [*et*](https://paperpile.com/c/71qymx/9WpW+c88U6) [*al.*](https://paperpile.com/c/71qymx/9WpW+c88U6) [*2014),*](https://paperpile.com/c/71qymx/9WpW+c88U6) *and the choice of regions has a great impact on model performance* [*(Jeong*](https://paperpile.com/c/71qymx/6l6cS) [*et*](https://paperpile.com/c/71qymx/6l6cS) [*al.*](https://paperpile.com/c/71qymx/6l6cS) [*2022)*](https://paperpile.com/c/71qymx/6l6cS)*. Common inclusion criteria include regions with adequate coverage, low intra-cell-type variance and high inter-cell-type methylation difference. Common exclusion criteria include regions susceptible to copy-number alterations and repeat elements. In addition to different deconvolution methods, this study compares several methods for marker selection, analyzes the performance of individual marker regions and shows that haplotype-aware deconvolution methods should use markers tailored for methylation haplotypes.”*

*Regarding your specific question, we provide some additional background and explanation below, but feel this is probably too much detail to add to the manuscript since it is discussed in the papers we cite:*

*Segmentation is the process of dividing the genome into smaller candidate regions, often considered a preliminary step before selecting marker regions. Segmentation determines the resolution of marker regions. At the most granular level, each CpG site is treated as an individual unit for analysis* [*(Aryee*](https://paperpile.com/c/71qymx/f9Qyd+ErhB7) [*et*](https://paperpile.com/c/71qymx/f9Qyd+ErhB7) [*al.*](https://paperpile.com/c/71qymx/f9Qyd+ErhB7) [*2014;*](https://paperpile.com/c/71qymx/f9Qyd+ErhB7) [*Park*](https://paperpile.com/c/71qymx/f9Qyd+ErhB7) [*and*](https://paperpile.com/c/71qymx/f9Qyd+ErhB7) [*Wu*](https://paperpile.com/c/71qymx/f9Qyd+ErhB7) [*2016)*](https://paperpile.com/c/71qymx/f9Qyd+ErhB7)*. Alternatively, clustering adjacent CpGs together can capture locally coordinated patterns of DNA methylation. However, this approach introduces the challenge of precisely defining the boundaries of each methylation block* [*(F*](https://paperpile.com/c/71qymx/QjLZ5+ViEt) [*et*](https://paperpile.com/c/71qymx/QjLZ5+ViEt) [*al.*](https://paperpile.com/c/71qymx/QjLZ5+ViEt) [*2016;*](https://paperpile.com/c/71qymx/QjLZ5+ViEt) [*Loyfer*](https://paperpile.com/c/71qymx/QjLZ5+ViEt) [*et*](https://paperpile.com/c/71qymx/QjLZ5+ViEt) [*al. 2023)*](https://paperpile.com/c/71qymx/QjLZ5+ViEt)*.*

*The marker selection process employed by Loyfer et al. is different from the Caggiano et al. approach. In the CelFiE package, segmentation is at the single CpG level. The CpGs are ranked by distance between methylation in the target cell type and the median methylation across all cell types. Then, the 100 best ranked CpGs per cell type are selected. A window of 500bp around each marker CpG serves as the final marker set. In contrast, the Loyfer segmentation includes multiple adjacent CpGs and aims to divide the genome into homogeneous blocks. The distance metric is also different, being the methylation difference between the second highest sample in the target cell type and the 75th percentile in all other samples. They published two region sets: the succinct U25, and an expanded U250 set, with 25 and 250 regions per cell type respectively.*

*We tested the performance of all deconvolution models on all three region sets. We saw deconvolution error changed depending on the marker set, and was generally higher with fewer marker regions. UXM performed better than CelFiE on U250, while CelFiE performed better on TIM regions.*

**Page 11, lines 30-31: The Epistate bias needs some expansion, why is the overestimation happening?**

*Response: We attribute this bias to too many degrees of freedom within the epistate model. In this model, both methylation states can potentially be present for every cell type, noted by a probability we term**. The assignment of a read to its cell type of origin relies on the prior probability of that cell type and the methylation pattern of the read. The odds of an abundant cell type displaying a rare pattern are* *and the odds of a rare cell type displaying its common pattern are**. The values of* *are estimated from the reference atlas and are often non-binary due to noise stemming from contamination or intra-cell type heterogeneity in the atlas. Frequently, the prior probability for common cell types becomes sufficiently large that even reads with rare methylation patterns are erroneously assigned to them.*

*One approach to address this issue is to binarize the* *values, thereby hard-assigning methylation states to cell types. When we applied this method to the U250 in silico mixtures, using the cell-type labels from Loyfer et al for binarization, we observed a significant reduction in bias:*

*However, with the binarized version we saw a contrasting phenomenon: an overestimation of rare cell types and an underestimation of abundant cell types. This occurs because the model becomes intolerant of* *heterogeneity in the background samples. This new figure is now included as supplementary Figure 4 in order to shed light on the issues with Epistate.*

*Overall, we did not find any configuration where the Epistate model outperformed other methods across different read lengths. We did consider removing Epistate from the manuscript entirely, but we decided that it would be helpful to the community to include it, since it is a model that has been proposed before, and others may want to use our open-source code to do additional research on this model. We believe further research could potentially identify an optimal penalty mechanism that allows for some flexibility in lambda without introducing the bias observed in this study.*

*We added the following text to provide a better explanation, along with the new Supplemental Figure:*

*“Unlike all other models, Epistate had a systematic bias that overestimated abundant cell types, and this bias was not improved with increasing genome coverage to 30x. We attribute this bias to too many degrees of freedom within the epistate model. In this model, both methylation states can potentially be present for every cell type. The assignment of a read to its cell type of origin relies on the prior probability of that cell type and the methylation pattern of the read. Frequently, the prior probability for common cell types becomes sufficiently large that even reads with rare methylation patterns are erroneously assigned to them. One approach is to make state assignments (lambdas) binary - this can decrease overall error but leads to overestimation of rare cell types (Fig S6). Overall, we did not find any configuration where the Epistate model outperformed other methods across different read lengths.”*

**Figure 3B: Please expand on these findings why the instability of a smaller space, again the error needs some context to interpret the error rate observed and the impact on the biological interpretation of the results.**

*Response: We observed a decrease in error with higher coverage. This is somewhat expected, as the availability of more high-quality fragments allows for more precise estimations.*

*We added this to the main text:*

*An analysis of error rates across a range of genome coverages (Figure 3B) verifies that error is lowest in the U250 marker set across all samples. As coverage increases, the error diminishes due to the lesser impact of outlier or ambiguous fragments. In order to ensure that specific cell types were not driving these results, we repeated all benchmarking on two additional random shufflings of cell type proportions (Figures S4-S5).*

**Figure 4E: I do not see a caption for this area of the figure, this needs to be explained better in the caption.**

*Response: Thank you for bringing this to our attention. The figure legend now reads: (E) Individual ROC curves for top 4 cell types, as ranked by AUC: erythrocyte progenitors, colon epithelium, pancreas and lung. The ROC curves display the predictive performance of the cell type fraction, as estimated by each model, in determining WHO ordinal code >= 7.*

**For broader adoption, it is important to provide a step by step process for the installation and some troubleshooting.**

*Response: A detailed tutorial was added to the github repository:* [*https://github.com/methylgrammarlab/deconvolution_models/blob/main/TUTORIAL.md*](https://github.com/methylgrammarlab/deconvolution_models/blob/main/TUTORIAL.md)

## Reviewer 2

**I do not have any major concerns. The simulations were well designed, and the benchmarking is quite rigorous. I have only one comment/suggestion: all the simulations were done based on WGBS data, and I don't have a big problem with that. However, in real world applications, such as the assays developed by Grail and Guardant Health, targeted bisulfite sequencing was used to increase the coverage of the informative markers and sensitivity under certain cost limits. Is it possible to add a simulation on targeted sequencing data, to find out how low they can go in terms of the limit of detection?**

*Response: Our deconvolution strategy for WGBS involves prioritizing informative markers while disregarding other genomic regions. Consequently, the accuracy of deconvolution is only influenced by the coverage of these informative regions. In fact, in all in silico mixtures, we only combined reads from these specific areas because any other reads would be unused. When marker regions are predefined, targeted sequencing can enhance their coverage within a given budget. This is specifically why we included coverage of 100X for the sensitivity benchmarking (Figure 4B-C). While 100X coverage is cost prohibitive for WGBS, it approximates coverage that can be achieved with capture panels such as GRAIL and Guardant. We added the following text to the Discussion section to point this out:*

*“While these 5x results envision shallow WGBS* [*(Katsman*](https://paperpile.com/c/71qymx/ARLi) [*et*](https://paperpile.com/c/71qymx/ARLi) [*al.*](https://paperpile.com/c/71qymx/ARLi) [*2022)*](https://paperpile.com/c/71qymx/ARLi)*, deconvolution can also used to analyze bisulfite capture-seq* [*(Klein*](https://paperpile.com/c/71qymx/hqFV) [*et*](https://paperpile.com/c/71qymx/hqFV) [*al.*](https://paperpile.com/c/71qymx/hqFV) [*2021)*](https://paperpile.com/c/71qymx/hqFV) *or Reduced Representation Bisulfite Sequencing* [*(Li*](https://paperpile.com/c/71qymx/Qimx) [*et*](https://paperpile.com/c/71qymx/Qimx) [*al.*](https://paperpile.com/c/71qymx/Qimx) [*2023)*](https://paperpile.com/c/71qymx/Qimx)*, where data for a specific set of genomic markers is collected at >100X depth to reduce cost. Our results for 100X sequencing using either the U250 set (Figure 4B-C) or a set of markers defined by RRBS (Figure S9), confirm that CelFiE-ISH outperformed other methods on cell fractions down to 0.03%.”*

*Based on your comment, we decided to also look for a publicly available capture dataset. We could not find any capture data that is openly available at the read level, but we did find an open dataset that used Reduced Representation Bisulfiste Sequencing, where enzymatic digestion and fragment size selection boost coverage of CpG-rich regions* [*(Li*](https://paperpile.com/c/71qymx/Qimx) [*et*](https://paperpile.com/c/71qymx/Qimx) [*al.*](https://paperpile.com/c/71qymx/Qimx) [*2023)*](https://paperpile.com/c/71qymx/Qimx)*. Notably, the authors of this RRBS study spun out a new liquid biopsy early cancer detection company based on RRBS*

*(*[*https://earlydx.com/team/)*](https://earlydx.com/team/)*, underscoring the relevance of this data type for commercial cost reduction.*

*We* *created simulated mixtures of human cell types from the RRBS study* [*(Li*](https://paperpile.com/c/71qymx/Qimx) [*et*](https://paperpile.com/c/71qymx/Qimx) [*al. 2023)*](https://paperpile.com/c/71qymx/Qimx)*. Similar to our other 100X spike in experiments, we mixed a background composed of white blood cells with a spike-in* of *heart (in this case, bulk heart tissue rather than sorted cardiomyoctes). We used the full 39 cell-type U250 WGBS atlas as reference for deconvolution, excluding sex chromosomes. Importantly, approximately only 50% of the U250 marker set is overlapped by RRBS-covered regions, so potentially we could produce an improved marker set including only RRBS-covered regions.*

*We summed the proportions of Hear-Fibro and Heart-Cardio as the estimated heart proportion. At high input proportions, we see underestimation of the “heart” component. This can be explained by the many cell types present in heart tissue we did not consider as part of the “heart” component: vascular endothelial cells, adipocytes, smooth muscle, lymphoid tissue etc.*

*At lower input proportions, we can see higher dispersion compared to the WGBS mixture, with less discriminative power, across all models. At full 100X coverage, we observe a significant difference between the spike-in and null mixtures at 0.3% for CelFiE (p=0.007) and UXM (p=0.027), and 0.03% for CelFiE-ISH (p=0.016). For CelFiE, this is consistent with the performance observed by Li et al. at 120X* [*(Li*](https://paperpile.com/c/71qymx/Qimx) [*et*](https://paperpile.com/c/71qymx/Qimx) [*al.*](https://paperpile.com/c/71qymx/Qimx) [*2023)*](https://paperpile.com/c/71qymx/Qimx)*. We observe higher performance for UXM compared to NNLS in the RRBS publication, which we attribute to superior marker site selection.*

*This new analysis is now included as supplementary figure 9, and we added the following text:*

*Targeted sequencing can be used to increase the coverage of informative markers and provide higher sensitivity for the same sequencing cost. We used a recently published human tissue reduced-representation bisulfite sequencing (RRBS) atlas* [*(Li*](https://paperpile.com/c/71qymx/Qimx) [*et*](https://paperpile.com/c/71qymx/Qimx) [*al.*](https://paperpile.com/c/71qymx/Qimx) [*2023)*](https://paperpile.com/c/71qymx/Qimx) *to create spike-in mixtures of heart tissue in a background of white blood cells, at 100X genome coverage, and used the Loyfer U250 atlas to deconvolute them (figure S8). Overall,we observed underestimation of the heart component at high input proportions. This can be explained by the multitude of cell types present in heart tissue that are not represented in the cell type reference atlas. CelFiE-ISH was able to distinguish 0.03% spike-in from the null mixture (p=0.016), while the threshold for CelFiE and UXM was 0.3%. This threshold is consistent with reported sensitivity for CelFiE on RRBS* [*(Li*](https://paperpile.com/c/71qymx/Qimx) [*et*](https://paperpile.com/c/71qymx/Qimx) [*al.*](https://paperpile.com/c/71qymx/Qimx) [*2023)*](https://paperpile.com/c/71qymx/Qimx)*.*

*The figure legend reads:*

***FigureS9:Spike-InmixturesofRRBStissuesamples.*** *Heart RRBS samples were mixed at varying proportions into a background of WBC RRBS samples, and deconvoluted with the WGBS cell-type atlas, on U250 regions. Heart spike in mixtures are shown in blue, compared to null mixtures with no heart reads (red points). Heart estimates across 50 replicates are shown as standard deviation bars. Both axes are in log-scale.*

*The methods section now includes:*

*Insilicomixtures-RRBStissuemixtures*

*Publicly available PAT files for 57 WBC tissue samples and 23 heart samples from Li et al.* [*(Li*](https://paperpile.com/c/71qymx/Qimx) [*et al.*](https://paperpile.com/c/71qymx/Qimx) [*2023)*](https://paperpile.com/c/71qymx/Qimx) *were downloaded, along with metadata, from GEO series* [*GSE233417.*](https://www.ncbi.nlm.nih.gov/geo/query/acc.cgi?acc=GSE233417) *Reads from the heart samples were mixed into a WBC background at 100X total coverage in the same process described above, in the following proportions: 0, 0.0003, 0.001, 0.003, 0.01, .03, 0.1. At each input proportion, 50 mixtures were generated, sampling randomly across all samples per tissue. Since this is an independent dataset, we did not use the 31 cell type atlas used above as a holdout training dataset. We constructed a reference atlas containing all 39 cell types described in Loyfer et al.* [*(Loyfer*](https://paperpile.com/c/71qymx/ViEt) [*et*](https://paperpile.com/c/71qymx/ViEt) [*al.*](https://paperpile.com/c/71qymx/ViEt) [*2023)*](https://paperpile.com/c/71qymx/ViEt)*, using all 205 samples (table S1), on U250 regions, excluding sex chromosomes, retrieved from Loyfer et al. supplementary table 4B*

[*(Loyfer*](https://paperpile.com/c/71qymx/ViEt) [*et*](https://paperpile.com/c/71qymx/ViEt) [*al.*](https://paperpile.com/c/71qymx/ViEt) [*2023)*](https://paperpile.com/c/71qymx/ViEt)*. Beta values for these regions are available at* [*https://zenodo.org/doi/10.5281/zenodo.10799745.*](https://zenodo.org/doi/10.5281/zenodo.10799745.T-test) *We considered the sum of Hear-Fibro and Heart-Cardio as the estimated heart proportion. To assess the discrimination between null mixtures and spike-in, we performed an independent sample T-test for the estimated heart proportion in the two groups per model.*

# *References cited in reviewer responses*

[*Aryee,*](http://paperpile.com/b/71qymx/f9Qyd) [*Martin*](http://paperpile.com/b/71qymx/f9Qyd) [*J.,*](http://paperpile.com/b/71qymx/f9Qyd) [*Andrew*](http://paperpile.com/b/71qymx/f9Qyd) [*E.*](http://paperpile.com/b/71qymx/f9Qyd) [*Jaffe,*](http://paperpile.com/b/71qymx/f9Qyd) [*Hector*](http://paperpile.com/b/71qymx/f9Qyd) [*Corrada-Bravo,*](http://paperpile.com/b/71qymx/f9Qyd) [*Christine*](http://paperpile.com/b/71qymx/f9Qyd) [*Ladd-Acosta, Andrew*](http://paperpile.com/b/71qymx/f9Qyd) [*P.*](http://paperpile.com/b/71qymx/f9Qyd) [*Feinberg,*](http://paperpile.com/b/71qymx/f9Qyd) [*Kasper*](http://paperpile.com/b/71qymx/f9Qyd) [*D.*](http://paperpile.com/b/71qymx/f9Qyd) [*Hansen,*](http://paperpile.com/b/71qymx/f9Qyd) [*and*](http://paperpile.com/b/71qymx/f9Qyd) [*Rafael*](http://paperpile.com/b/71qymx/f9Qyd) [*A.*](http://paperpile.com/b/71qymx/f9Qyd) [*Irizarry.*](http://paperpile.com/b/71qymx/f9Qyd) [*2014.*](http://paperpile.com/b/71qymx/f9Qyd) [*“Minfi:*](http://paperpile.com/b/71qymx/f9Qyd) [*A Flexible*](http://paperpile.com/b/71qymx/f9Qyd) [*and*](http://paperpile.com/b/71qymx/f9Qyd) [*Comprehensive*](http://paperpile.com/b/71qymx/f9Qyd) [*Bioconductor*](http://paperpile.com/b/71qymx/f9Qyd) [*Package*](http://paperpile.com/b/71qymx/f9Qyd) [*for*](http://paperpile.com/b/71qymx/f9Qyd) [*the*](http://paperpile.com/b/71qymx/f9Qyd) [*Analysis*](http://paperpile.com/b/71qymx/f9Qyd) [*of*](http://paperpile.com/b/71qymx/f9Qyd) [*Infinium DNA*](http://paperpile.com/b/71qymx/f9Qyd) [*Methylation*](http://paperpile.com/b/71qymx/f9Qyd) [*Microarrays.”*](http://paperpile.com/b/71qymx/f9Qyd) [*Bioinformatics*](http://paperpile.com/b/71qymx/f9Qyd) [*30*](http://paperpile.com/b/71qymx/f9Qyd) [*(10):*](http://paperpile.com/b/71qymx/f9Qyd) [*1363–69.*](http://paperpile.com/b/71qymx/f9Qyd)

[*Caggiano,*](http://paperpile.com/b/71qymx/9WpW) [*Christa,*](http://paperpile.com/b/71qymx/9WpW) [*Barbara*](http://paperpile.com/b/71qymx/9WpW) [*Celona,*](http://paperpile.com/b/71qymx/9WpW) [*Fleur*](http://paperpile.com/b/71qymx/9WpW) [*Garton,*](http://paperpile.com/b/71qymx/9WpW) [*Joel*](http://paperpile.com/b/71qymx/9WpW) [*Mefford,*](http://paperpile.com/b/71qymx/9WpW) [*Brian*](http://paperpile.com/b/71qymx/9WpW) [*L.*](http://paperpile.com/b/71qymx/9WpW) [*Black,*](http://paperpile.com/b/71qymx/9WpW) [*Robert Henderson,*](http://paperpile.com/b/71qymx/9WpW) [*Catherine*](http://paperpile.com/b/71qymx/9WpW) [*Lomen-Hoerth,*](http://paperpile.com/b/71qymx/9WpW) [*Andrew*](http://paperpile.com/b/71qymx/9WpW) [*Dahl,*](http://paperpile.com/b/71qymx/9WpW) [*and*](http://paperpile.com/b/71qymx/9WpW) [*Noah*](http://paperpile.com/b/71qymx/9WpW) [*Zaitlen.*](http://paperpile.com/b/71qymx/9WpW) [*2021. “Comprehensive*](http://paperpile.com/b/71qymx/9WpW) [*Cell*](http://paperpile.com/b/71qymx/9WpW) [*Type*](http://paperpile.com/b/71qymx/9WpW) [*Decomposition*](http://paperpile.com/b/71qymx/9WpW) [*of*](http://paperpile.com/b/71qymx/9WpW) [*Circulating*](http://paperpile.com/b/71qymx/9WpW) [*Cell-Free*](http://paperpile.com/b/71qymx/9WpW) [*DNA*](http://paperpile.com/b/71qymx/9WpW) [*with CelFiE.”*](http://paperpile.com/b/71qymx/9WpW) [*Nature*](http://paperpile.com/b/71qymx/9WpW) [*Communications*](http://paperpile.com/b/71qymx/9WpW) [*2021*](http://paperpile.com/b/71qymx/9WpW) [*12:1*](http://paperpile.com/b/71qymx/9WpW) [*12*](http://paperpile.com/b/71qymx/9WpW) [*(1):*](http://paperpile.com/b/71qymx/9WpW) [*1–13.*](http://paperpile.com/b/71qymx/9WpW)

[*F,*](http://paperpile.com/b/71qymx/QjLZ5) [*Jühling,*](http://paperpile.com/b/71qymx/QjLZ5) [*Kretzmer*](http://paperpile.com/b/71qymx/QjLZ5) [*H,*](http://paperpile.com/b/71qymx/QjLZ5) [*Bernhart*](http://paperpile.com/b/71qymx/QjLZ5) [*Sh,*](http://paperpile.com/b/71qymx/QjLZ5) [*Otto*](http://paperpile.com/b/71qymx/QjLZ5) [*C,*](http://paperpile.com/b/71qymx/QjLZ5) [*Stadler*](http://paperpile.com/b/71qymx/QjLZ5) [*Pf,*](http://paperpile.com/b/71qymx/QjLZ5) [*and*](http://paperpile.com/b/71qymx/QjLZ5) [*Hoffmann*](http://paperpile.com/b/71qymx/QjLZ5) [*S.*](http://paperpile.com/b/71qymx/QjLZ5) [*2016. “Metilene:*](http://paperpile.com/b/71qymx/QjLZ5) [*Fast*](http://paperpile.com/b/71qymx/QjLZ5) [*and*](http://paperpile.com/b/71qymx/QjLZ5) [*Sensitive*](http://paperpile.com/b/71qymx/QjLZ5) [*Calling*](http://paperpile.com/b/71qymx/QjLZ5) [*of*](http://paperpile.com/b/71qymx/QjLZ5) [*Differentially*](http://paperpile.com/b/71qymx/QjLZ5) [*Methylated*](http://paperpile.com/b/71qymx/QjLZ5) [*Regions*](http://paperpile.com/b/71qymx/QjLZ5) [*from Bisulfite*](http://paperpile.com/b/71qymx/QjLZ5) [*Sequencing*](http://paperpile.com/b/71qymx/QjLZ5) [*Data.”*](http://paperpile.com/b/71qymx/QjLZ5) [*Genome*](http://paperpile.com/b/71qymx/QjLZ5) [*Research*](http://paperpile.com/b/71qymx/QjLZ5) [*26*](http://paperpile.com/b/71qymx/QjLZ5) [*(2):*](http://paperpile.com/b/71qymx/QjLZ5) [*256–62.*](http://paperpile.com/b/71qymx/QjLZ5)

[*Jeong,*](http://paperpile.com/b/71qymx/6l6cS) [*Yunhee,*](http://paperpile.com/b/71qymx/6l6cS) [*Lisa*](http://paperpile.com/b/71qymx/6l6cS) [*Barros*](http://paperpile.com/b/71qymx/6l6cS) [*de*](http://paperpile.com/b/71qymx/6l6cS) [*Andrade*](http://paperpile.com/b/71qymx/6l6cS) [*E*](http://paperpile.com/b/71qymx/6l6cS) [*Sousa,*](http://paperpile.com/b/71qymx/6l6cS) [*Dominik*](http://paperpile.com/b/71qymx/6l6cS) [*Thalmeier,*](http://paperpile.com/b/71qymx/6l6cS) [*Reka*](http://paperpile.com/b/71qymx/6l6cS) [*Toth, Marlene*](http://paperpile.com/b/71qymx/6l6cS) [*Ganslmeier,*](http://paperpile.com/b/71qymx/6l6cS) [*Kersten*](http://paperpile.com/b/71qymx/6l6cS) [*Breuer,*](http://paperpile.com/b/71qymx/6l6cS) [*Christoph*](http://paperpile.com/b/71qymx/6l6cS) [*Plass,*](http://paperpile.com/b/71qymx/6l6cS) [*and*](http://paperpile.com/b/71qymx/6l6cS) [*Pavlo*](http://paperpile.com/b/71qymx/6l6cS) [*Lutsik.*](http://paperpile.com/b/71qymx/6l6cS) [*2022. “Systematic*](http://paperpile.com/b/71qymx/6l6cS) [*Evaluation*](http://paperpile.com/b/71qymx/6l6cS) [*of*](http://paperpile.com/b/71qymx/6l6cS) [*Cell-Type*](http://paperpile.com/b/71qymx/6l6cS) [*Deconvolution*](http://paperpile.com/b/71qymx/6l6cS) [*Pipelines*](http://paperpile.com/b/71qymx/6l6cS) [*for*](http://paperpile.com/b/71qymx/6l6cS) [*Sequencing-Based Bulk*](http://paperpile.com/b/71qymx/6l6cS) [*DNA*](http://paperpile.com/b/71qymx/6l6cS) [*Methylomes.”*](http://paperpile.com/b/71qymx/6l6cS) [*Briefings*](http://paperpile.com/b/71qymx/6l6cS) [*in*](http://paperpile.com/b/71qymx/6l6cS) [*Bioinformatics*](http://paperpile.com/b/71qymx/6l6cS) [*23*](http://paperpile.com/b/71qymx/6l6cS) [*(4).*](http://paperpile.com/b/71qymx/6l6cS)

[*https://doi.org*](http://paperpile.com/b/71qymx/6l6cS)[*/10.1093/bib/bbac24*](http://dx.doi.org/10.1093/bib/bbac248)[*8.*](http://paperpile.com/b/71qymx/6l6cS)

[*Katsman,*](http://paperpile.com/b/71qymx/ARLi) [*Efrat,*](http://paperpile.com/b/71qymx/ARLi) [*Shari*](http://paperpile.com/b/71qymx/ARLi) [*Orlanski,*](http://paperpile.com/b/71qymx/ARLi) [*Filippo*](http://paperpile.com/b/71qymx/ARLi) [*Martignano,*](http://paperpile.com/b/71qymx/ARLi) [*Ilana*](http://paperpile.com/b/71qymx/ARLi) [*Fox-Fisher,*](http://paperpile.com/b/71qymx/ARLi) [*Ruth*](http://paperpile.com/b/71qymx/ARLi) [*Shemer, Yuval*](http://paperpile.com/b/71qymx/ARLi) [*Dor,*](http://paperpile.com/b/71qymx/ARLi) [*Aviad*](http://paperpile.com/b/71qymx/ARLi) [*Zick,*](http://paperpile.com/b/71qymx/ARLi) [*et*](http://paperpile.com/b/71qymx/ARLi) [*al.*](http://paperpile.com/b/71qymx/ARLi) [*2022.*](http://paperpile.com/b/71qymx/ARLi) [*“Detecting*](http://paperpile.com/b/71qymx/ARLi) [*Cell-of-Origin*](http://paperpile.com/b/71qymx/ARLi) [*and*](http://paperpile.com/b/71qymx/ARLi) [*Cancer-Specific Methylation*](http://paperpile.com/b/71qymx/ARLi) [*Features*](http://paperpile.com/b/71qymx/ARLi) [*of*](http://paperpile.com/b/71qymx/ARLi) [*Cell-Free*](http://paperpile.com/b/71qymx/ARLi) [*DNA*](http://paperpile.com/b/71qymx/ARLi) [*from*](http://paperpile.com/b/71qymx/ARLi) [*Nanopore*](http://paperpile.com/b/71qymx/ARLi) [*Sequencing.”*](http://paperpile.com/b/71qymx/ARLi) [*Genome Biology*](http://paperpile.com/b/71qymx/ARLi) [*23*](http://paperpile.com/b/71qymx/ARLi) [*(1):*](http://paperpile.com/b/71qymx/ARLi) [*158.*](http://paperpile.com/b/71qymx/ARLi)

[*Klein,*](http://paperpile.com/b/71qymx/hqFV) [*E.*](http://paperpile.com/b/71qymx/hqFV) [*A.,*](http://paperpile.com/b/71qymx/hqFV) [*D.*](http://paperpile.com/b/71qymx/hqFV) [*Richards,*](http://paperpile.com/b/71qymx/hqFV) [*A.*](http://paperpile.com/b/71qymx/hqFV) [*Cohn,*](http://paperpile.com/b/71qymx/hqFV) [*M.*](http://paperpile.com/b/71qymx/hqFV) [*Tummala,*](http://paperpile.com/b/71qymx/hqFV) [*R.*](http://paperpile.com/b/71qymx/hqFV) [*Lapham,*](http://paperpile.com/b/71qymx/hqFV) [*D.*](http://paperpile.com/b/71qymx/hqFV) [*Cosgrove,*](http://paperpile.com/b/71qymx/hqFV) [*G.*](http://paperpile.com/b/71qymx/hqFV) [*Chung,*](http://paperpile.com/b/71qymx/hqFV) [*et al.*](http://paperpile.com/b/71qymx/hqFV) [*2021.*](http://paperpile.com/b/71qymx/hqFV) [*“Clinical*](http://paperpile.com/b/71qymx/hqFV) [*Validation*](http://paperpile.com/b/71qymx/hqFV) [*of*](http://paperpile.com/b/71qymx/hqFV) [*a*](http://paperpile.com/b/71qymx/hqFV) [*Targeted*](http://paperpile.com/b/71qymx/hqFV) [*Methylation-Based*](http://paperpile.com/b/71qymx/hqFV) [*Multi-Cancer*](http://paperpile.com/b/71qymx/hqFV) [*Early Detection*](http://paperpile.com/b/71qymx/hqFV) [*Test*](http://paperpile.com/b/71qymx/hqFV) [*Using*](http://paperpile.com/b/71qymx/hqFV) [*an*](http://paperpile.com/b/71qymx/hqFV) [*Independent*](http://paperpile.com/b/71qymx/hqFV) [*Validation*](http://paperpile.com/b/71qymx/hqFV) [*Set.”*](http://paperpile.com/b/71qymx/hqFV) [*Annals*](http://paperpile.com/b/71qymx/hqFV) [*of*](http://paperpile.com/b/71qymx/hqFV) [*Oncology:*](http://paperpile.com/b/71qymx/hqFV) [*Official Journal*](http://paperpile.com/b/71qymx/hqFV) [*of*](http://paperpile.com/b/71qymx/hqFV) [*the*](http://paperpile.com/b/71qymx/hqFV) [*European*](http://paperpile.com/b/71qymx/hqFV) [*Society*](http://paperpile.com/b/71qymx/hqFV) [*for*](http://paperpile.com/b/71qymx/hqFV) [*Medical*](http://paperpile.com/b/71qymx/hqFV) [*Oncology*](http://paperpile.com/b/71qymx/hqFV) [*/*](http://paperpile.com/b/71qymx/hqFV) [*ESMO*](http://paperpile.com/b/71qymx/hqFV) [*32*](http://paperpile.com/b/71qymx/hqFV) [*(9):*](http://paperpile.com/b/71qymx/hqFV) [*1167–77.*](http://paperpile.com/b/71qymx/hqFV)

[*Li,*](http://paperpile.com/b/71qymx/Qimx) [*Shuo,*](http://paperpile.com/b/71qymx/Qimx) [*Weihua*](http://paperpile.com/b/71qymx/Qimx) [*Zeng,*](http://paperpile.com/b/71qymx/Qimx) [*Xiaohui*](http://paperpile.com/b/71qymx/Qimx) [*Ni,*](http://paperpile.com/b/71qymx/Qimx) [*Qiao*](http://paperpile.com/b/71qymx/Qimx) [*Liu,*](http://paperpile.com/b/71qymx/Qimx) [*Wenyuan*](http://paperpile.com/b/71qymx/Qimx) [*Li,*](http://paperpile.com/b/71qymx/Qimx) [*Mary*](http://paperpile.com/b/71qymx/Qimx) [*L.*](http://paperpile.com/b/71qymx/Qimx) [*Stackpole,*](http://paperpile.com/b/71qymx/Qimx) [*Yonggang Zhou,*](http://paperpile.com/b/71qymx/Qimx) [*et*](http://paperpile.com/b/71qymx/Qimx) [*al.*](http://paperpile.com/b/71qymx/Qimx) [*2023.*](http://paperpile.com/b/71qymx/Qimx) [*“Comprehensive*](http://paperpile.com/b/71qymx/Qimx) [*Tissue*](http://paperpile.com/b/71qymx/Qimx) [*Deconvolution*](http://paperpile.com/b/71qymx/Qimx) [*of*](http://paperpile.com/b/71qymx/Qimx) [*Cell-Free*](http://paperpile.com/b/71qymx/Qimx) [*DNA*](http://paperpile.com/b/71qymx/Qimx) [*by*](http://paperpile.com/b/71qymx/Qimx)

[*Deep*](http://paperpile.com/b/71qymx/Qimx) [*Learning*](http://paperpile.com/b/71qymx/Qimx) [*for*](http://paperpile.com/b/71qymx/Qimx) [*Disease*](http://paperpile.com/b/71qymx/Qimx) [*Diagnosis*](http://paperpile.com/b/71qymx/Qimx) [*and*](http://paperpile.com/b/71qymx/Qimx) [*Monitoring.”*](http://paperpile.com/b/71qymx/Qimx) [*Proceedings*](http://paperpile.com/b/71qymx/Qimx) [*of*](http://paperpile.com/b/71qymx/Qimx) [*the*](http://paperpile.com/b/71qymx/Qimx) [*National Academy*](http://paperpile.com/b/71qymx/Qimx) [*of*](http://paperpile.com/b/71qymx/Qimx) [*Sciences*](http://paperpile.com/b/71qymx/Qimx) [*of*](http://paperpile.com/b/71qymx/Qimx) [*the*](http://paperpile.com/b/71qymx/Qimx) [*United*](http://paperpile.com/b/71qymx/Qimx) [*States*](http://paperpile.com/b/71qymx/Qimx) [*of*](http://paperpile.com/b/71qymx/Qimx) [*America*](http://paperpile.com/b/71qymx/Qimx) [*120*](http://paperpile.com/b/71qymx/Qimx) [*(28):*](http://paperpile.com/b/71qymx/Qimx) [*e2305236120.*](http://paperpile.com/b/71qymx/Qimx)

[*Loyfer,*](http://paperpile.com/b/71qymx/ViEt) [*Netanel,*](http://paperpile.com/b/71qymx/ViEt) [*Judith*](http://paperpile.com/b/71qymx/ViEt) [*Magenheim,*](http://paperpile.com/b/71qymx/ViEt) [*Ayelet*](http://paperpile.com/b/71qymx/ViEt) [*Peretz,*](http://paperpile.com/b/71qymx/ViEt) [*Gordon*](http://paperpile.com/b/71qymx/ViEt) [*Cann,*](http://paperpile.com/b/71qymx/ViEt) [*Joerg*](http://paperpile.com/b/71qymx/ViEt) [*Bredno,*](http://paperpile.com/b/71qymx/ViEt) [*Agnes Klochendler,*](http://paperpile.com/b/71qymx/ViEt) [*Ilana*](http://paperpile.com/b/71qymx/ViEt) [*Fox-Fisher,*](http://paperpile.com/b/71qymx/ViEt) [*et*](http://paperpile.com/b/71qymx/ViEt) [*al.*](http://paperpile.com/b/71qymx/ViEt) [*2023.*](http://paperpile.com/b/71qymx/ViEt) [*“A*](http://paperpile.com/b/71qymx/ViEt) [*DNA*](http://paperpile.com/b/71qymx/ViEt) [*Methylation*](http://paperpile.com/b/71qymx/ViEt) [*Atlas*](http://paperpile.com/b/71qymx/ViEt) [*of*](http://paperpile.com/b/71qymx/ViEt) [*Normal Human*](http://paperpile.com/b/71qymx/ViEt) [*Cell*](http://paperpile.com/b/71qymx/ViEt) [*Types.”*](http://paperpile.com/b/71qymx/ViEt) [*Nature,*](http://paperpile.com/b/71qymx/ViEt) [*January,*](http://paperpile.com/b/71qymx/ViEt) [*1–10.*](http://paperpile.com/b/71qymx/ViEt)

[*Park,*](http://paperpile.com/b/71qymx/ErhB7) [*Yongseok,*](http://paperpile.com/b/71qymx/ErhB7) [*and*](http://paperpile.com/b/71qymx/ErhB7) [*Hao*](http://paperpile.com/b/71qymx/ErhB7) [*Wu.*](http://paperpile.com/b/71qymx/ErhB7) [*2016.*](http://paperpile.com/b/71qymx/ErhB7) [*“Differential*](http://paperpile.com/b/71qymx/ErhB7) [*Methylation*](http://paperpile.com/b/71qymx/ErhB7) [*Analysis*](http://paperpile.com/b/71qymx/ErhB7) [*for*](http://paperpile.com/b/71qymx/ErhB7) [*BS-Seq*](http://paperpile.com/b/71qymx/ErhB7) [*Data under*](http://paperpile.com/b/71qymx/ErhB7) [*General*](http://paperpile.com/b/71qymx/ErhB7) [*Experimental*](http://paperpile.com/b/71qymx/ErhB7) [*Design.”*](http://paperpile.com/b/71qymx/ErhB7) [*Bioinformatics*](http://paperpile.com/b/71qymx/ErhB7) [*32*](http://paperpile.com/b/71qymx/ErhB7) [*(10):*](http://paperpile.com/b/71qymx/ErhB7) [*1446–53.*](http://paperpile.com/b/71qymx/ErhB7)

[*Zheng,*](http://paperpile.com/b/71qymx/c88U6) [*Xiaoqi,*](http://paperpile.com/b/71qymx/c88U6) [*Qian*](http://paperpile.com/b/71qymx/c88U6) [*Zhao,*](http://paperpile.com/b/71qymx/c88U6) [*Hua-Jun*](http://paperpile.com/b/71qymx/c88U6) [*Wu,*](http://paperpile.com/b/71qymx/c88U6) [*Wei*](http://paperpile.com/b/71qymx/c88U6) [*Li,*](http://paperpile.com/b/71qymx/c88U6) [*Haiyun*](http://paperpile.com/b/71qymx/c88U6) [*Wang,*](http://paperpile.com/b/71qymx/c88U6) [*Clifford*](http://paperpile.com/b/71qymx/c88U6) [*A.*](http://paperpile.com/b/71qymx/c88U6) [*Meyer,*](http://paperpile.com/b/71qymx/c88U6) [*Qian Alvin*](http://paperpile.com/b/71qymx/c88U6) [*Qin,*](http://paperpile.com/b/71qymx/c88U6) [*et*](http://paperpile.com/b/71qymx/c88U6) [*al.*](http://paperpile.com/b/71qymx/c88U6) [*2014.*](http://paperpile.com/b/71qymx/c88U6) [*“MethylPurify:*](http://paperpile.com/b/71qymx/c88U6) [*Tumor*](http://paperpile.com/b/71qymx/c88U6) [*Purity*](http://paperpile.com/b/71qymx/c88U6) [*Deconvolution*](http://paperpile.com/b/71qymx/c88U6) [*and*](http://paperpile.com/b/71qymx/c88U6) [*Differential Methylation*](http://paperpile.com/b/71qymx/c88U6) [*Detection*](http://paperpile.com/b/71qymx/c88U6) [*from*](http://paperpile.com/b/71qymx/c88U6) [*Single*](http://paperpile.com/b/71qymx/c88U6) [*Tumor*](http://paperpile.com/b/71qymx/c88U6) [*DNA*](http://paperpile.com/b/71qymx/c88U6) [*Methylomes.”*](http://paperpile.com/b/71qymx/c88U6) [*Genome*](http://paperpile.com/b/71qymx/c88U6) [*Biology*](http://paperpile.com/b/71qymx/c88U6) [*2014 15:7*](http://paperpile.com/b/71qymx/c88U6) [*15*](http://paperpile.com/b/71qymx/c88U6) [*(7):*](http://paperpile.com/b/71qymx/c88U6) [*1–13.*](http://paperpile.com/b/71qymx/c88U6)

**Second round of review**

**Reviewer 1**

The authors have answered all my questions and provided a very comprehensive revised manuscript.

**Reviewer 2**

The authors have fully addressed my concerns. I'm supportive of accepting this manuscript.
